# Supplementary material for: Structural and functional fine mapping of cysteines in mammalian glutaredoxin reveal their differential oxidation susceptibility
Source: Nat Commun. 2023 Jul 28;14:4550. doi: 10.1038/s41467-023-39664-2 (PMC10382592; doi:10.1038/s41467-023-39664-2)

Supplementary Information

Methods

Bacterial Transformation with GLRX Plasmids

BL21(DE3) competent cells (Agilent) were transformed with 20 ng of GLRX1 wild type or mutant plasmids according to the manufacturer's directions. Briefly, 50 µL of competent cells treated with 0.85 µL β-mercaptoethanol on ice for 10 min, followed by incubation with GLRX-1 plasmid on ice for 30 min and heat shocked for 45 sec in a 42°C water bath. After returning to ice for 2 min, 900 µL of prewarmed (42°C) S.O.C. media (Thermo Fisher Scientific, Waltham, MA) was added and samples were placed on a shaker at 22 g at 37°C for 1 hr. 100 µL of cells were streaked onto LB agar plates containing ampicillin (50µg/mL) and colonies were grown overnight at 37°C. A starter culture of one colony from each mutant was grown in 30 mL LB media with ampicillin (50µg/mL) and shaken for 6-8 hr at 300 rpm at 37°C. Resulting bacterial growth was added to 1 L of Overnight Express™ Instant TB Medium (MillipreSigma, Burlington, MA) containing ampicillin (50µg/mL) and shaken at 22 g at 37°C overnight. Bacterial cell pellets were collected by centrifuging cultures in 250mL collection bottles at 5,500 g at 4°C for 10 min. Supernatant was discarded and resulting pellets were purified or frozen at -80°C to be purified later.

Liquid chromatography-tandem mass spectrometry (LC-MS/MS)-based protein identification

The tryptic peptides resuspended in 2.5% acetonitrile (CAN) and 2.5% formic acid (FA) in water were analyzed on the Q-Exactive Plus mass spectrometer coupled to an EASY-nLC 1200 system (Thermo Fisher Scientific). Samples were loaded onto a 100 µm i.d. capillary column packed with UChrom C18 (1.8 µm particle size, 120 Å, Cat. No: PN-80001; Nanolcms, CA) at a flow rate of 300 nl min^{-1}. The column end was laser pulled to a ~3 µm orifice and packed with minimal amounts of 5um Magic C18AQ before packing with the 1.8 um particle size chromatographic materials. Peptides were separated by a solvent system composed of solvent A: 100% water/0.1% FA and solvent B: 80% ACN/0.1% FA with a gradient of 0-44% B over 60 min (for GLRX samples with

multiple mutations) 0-44% B over 150 min (for glutathionylated GLRX and single mutation GLRX samples), 44-100% B in 1 min and then 100% B for 8 min, followed by an immediate return to 100% A and a hold at 100% A for 20 min before the next injection. The GLRX samples with multiple mutations were randomized in run order and peptide standards (bovine serum albumin) were run between samples. Mass spectrometry data was acquired in a data-dependent "Top 10" acquisition mode with lock mass function activated (*m/z* 371.1012; use lock masses: best; lock mass injection: full MS), in which a survey scan from *m/z* 350-1600 at 70, 000 resolution (AGC target $1e^6$; max IT 100 ms; profile mode) was followed by 10 higher-energy collisional dissociation (HCD) tandem mass spectrometry (MS/MS) scans on the most abundant ions at 17,500 resolution (loop count = 10; AGC target $5e^4$; max IT 100 ms; centroid mode). MS/MS scans were acquired with an isolation width of 1.6 *m/z* and a normalized collisional energy of 26%. Dynamic exclusion was enabled (peptide match: preferred; exclude isotopes: on; underfill ratio: 1%; exclusion duration: 15 sec). Minimum AGC target = 5 e2.

Quantification of glutathionylation in GLRX

For quantifying glutathionylated GLRX peptides (two independent experiments), the search files (.msf) together with the raw files were imported into Skyline for selecting precursors or transitions for quantitation. Abundance of the GLRX peptides was determined by extracting the target ion chromatograms (extracted ion chromatograms: XICs) and determining the areas under the Satvisky Golay-smoothed ion elution profiles using "Peak Areas/Replicate Comparison" (Supplementary Table 10 and 11). Default parameters for peptide and transition settings were used with the following modifications. Peptide Settings: 1) Max missed cleavages = 2; 2) Max variable mods: = 4. Transition settings: 1) resolution and mass tolerance were set to match the Orbitrap settings (MS resolution: 70,000, MSMS resolution: 17,500 (PRM), Method/Ion match tolerance m/z: 0.05 Da), 2) Ion type: p (DDA) and p, b, y (PRM), 3) Precursor charges = 1, 2, 3 (DDA), and Ion charges = 1, 2 (PRM), and 4) MS1 filtering: Peaks = 1 and Precursor mass

analyzer set as “Orbitrap”. Various versions of the glutathionylated peptides with different combinations of modifications (CAM, GSH and SO3 on cysteines and methionine oxidation) were created via “modify …” to the target peptides. After setting the above-mentioned parameters, the raw files and search results were re-imported through “Manage Results”. The XICs were pinpointed by “Auto-Zoom”, and the retention times of the displayed XICs were compared with those determined by Proteome Discoverer for the elution profiles of the peptides of interest identified with the highest XCorr value. Boundaries of integration were manually evaluated and adjusted according to the elution profiles. Additional quantification by PRM was performed on the closely eluting VVVFIKPTC(GSH)PYC(O3)R and VVVFIKPTC(O3)PYC(GSH)R peptides. The abundance value was determined by summing the area of the XIC of the 3 transitions that are unique to the peptides (VVVFIKPTC(GSSG)PYC(O3)R: *m/z* 939.433 ++ → 326.113 $[y2]^+$,C(GSH), 489.176 $[y3]^+$ Y, and 586.229 $[y4]^+$ P, and VVFIKPTC(O3)PYC(GSH)R: ++ → 538.196 $[y2]^+$C(O3), 746.260 $[y3]^+$ Y, and 843.312 $[y4]^+$ P). The transitions were selected via “Pick Children”. The chromatographic traces were exported from Skyline to GraphPad Prism 8 for chromatogram plotting. The spectra with the highest XCorr value acquired during peak elution are included in the Supplementary Figure 3.

MD simulation on GLRX

As described in the method section, all the molecular dynamic simulations have been performed in the Desmond program in the SPC water box with the OPL3e force field for the protein. Supplementary Table 3 summarizes the parameters used in MD simulations.

MD Simulation of GLRX Multimers

The MD simulation of GLRX dimer (monomeric conformation PDBID: 4RQR) aligned to zebrafish GLRX (PDBID: 3UIW) represents the symmetrical C8-C8 interface (shown in surface mode on Supplementary Figure 8) which is driven mainly by polar interactions. In addition, three GLRX

monomers (PDBID: 4RQR) were arbitrarily placed in the simulation box with a separation over 5 Å. All the models were built in Maestro (Schrödinger, Inc.) and prepared for simulation in System Builder. The multistage equilibrium strategy in the Desmond package was used.

Predicted pK_a values of Cysteine residues by PROPKA

With the Amber18 program [62, 63], the protein model underwent minimization and equilibration in explicit solvent before *p*K_a calculations. First, the protein model was prepared in CHARMM-GUI [64] in explicit solvent model with a cubic box (66 Å) and a total of 27,535 atoms using the TIP3P water model and ff14SB force field for the protein. The system went through 5000 steps of steepest-descent minimization with a nonbonded cutoff of 9.0 Å, followed by 200 ns NPT simulation (300 K) using Langevin dynamics and applying constrain bonds containing hydrogens and time step of 0.002 ps. Finally, the *p*K_a calculation is performed as described in the main paper. Exploring the relative *p*K_a values of cysteines gives a deep understanding of their redox behavior in the solution. According to the predictions, C23 exhibits the highest tendency to lose proton (most acidic cysteine residue) followed by C8 (Supplementary Figure 6).

Induced-fit docking of GSSG to GLRX

All the protein-ligand dockings have been carried out on “Induced fit docking” package in Maestro 2019-2 [60]. The conformational sampling with energy window of 2.5 kcal/mol, receptor and ligand van der Waals scaling of 0.5, Cv_cutoff = 100 and Ligand_ccut of 0.15 have been applied.

Due to the polar nature of GSSG, it tends to interact with polar regions of the protein. In the case of C83, residues which mediate the GSSG bonding are R28, I32, V87 and S84 while C8, L43, E44, K9, Q11, I10 are involved when we docked GSSG to C8 (Supplementary Figure 9).

Quantum mechanics calculation:

A simple model compound has been selected for initial guess of the cysteines in the protein (Supplementary Figure 10). The IRC (intrinsic reaction coordinate) demonstrates that after passing the transition state the proton of the oxygen (O1) close to sulfur is transferred to other oxygen (O2) leading to formation of a water molecule (Supplementary Figure 4). The IRC has been performed in Maestro (2019) using B3LYP/6-31G* level of theory and leading to an E_a of 25 kcal/mol and satisfactorily reproduces the geometry of reactant state and product state. The free energy on the y-axis is relative gas phase energy to reactant state which is set to zero.

Coordinates of the minimized structures

Model compound:

Transition state:

final geometry:

angstroms

atom	x	y	z
C1	-2.5353574584	1.1868566320	-0.1765815290
S2	-1.0152126036	1.3900549656	0.8186836910
H3	-2.7987466316	0.1247327302	-0.2816016395
H4	-3.3844464594	1.7082131207	0.2840006960
H5	-2.3923190561	1.6141235225	-1.1761299933
O6	-1.7718178208	5.4193291511	0.1794789178
H7	-2.0704955795	5.1587029986	-0.7065821501
O8	-1.2090602859	3.5841204503	0.4167954224
H9	-1.6896706777	3.7246855044	1.2440305987

Reactant state:

final geometry:

angstroms

atom	x	y	z
C1	-0.9010157607	2.6219809773	-1.0608307349
S2	-2.6158764259	1.9336295997	-1.0279761176
H3	-0.6103858830	2.9735627768	-0.0636339442
H4	-0.1664941833	1.8699037460	-1.3793497129
H5	-0.8213033456	3.4715065750	-1.7528672372
O6	-2.5495318134	2.9770221847	1.9128584131
H7	-2.8232226816	2.9682985935	0.9525190403
O8	-2.1764285214	1.5624338821	2.0554649484
H9	-2.2520796239	1.2867760129	1.0985160029

Product state:

final geometry:

atom	x	y	z
	angstroms		
C1	-1.7794420394	1.2446928065	-0.2962944438
S2	-1.3779655147	1.7758780109	1.4217501725
H3	-1.5156413892	0.1865624557	-0.4366809293
H4	-2.8459774396	1.3810002685	-0.5238123819
H5	-1.1931237215	1.8697557058	-0.9796835210
O6	0.2457864401	3.9753399221	-0.2936805140
H7	-0.5292203264	3.8730636413	0.3685857310
O8	-1.6608182489	3.3766203399	1.3899798174
H9	0.7821590047	3.2242669537	-0.0092731293

QM/MM calculations of oxidation of GLRX

Over the course of reaction with H_2O_2, the complex may undergo significant charge redistribution with the negative charge of the sulfur atom pulled away by the oxygens on H_2O_2. The presence of polar interactions with the neighboring residues described below can further assist the transformation process in some cysteines compared to the model compound (see the methods section and the supplementary information for additional computational details as well as the optimized coordinates of the model $[CH_3S\cdots HOOH]^-$ transition state complex). At the transition state, the O–O bond is elongated, while the S–O distance is shortened compared to the S···O distance in the starting material complex (Supplementary Figure 10, Supplementary Table 6). The transition state coordinates as well as the intrinsic reaction coordinate plot (Supplementary Figure 4) demonstrate that a proton transfer from the oxygen closest to the sulfur atom (O1) to the second oxygen (O2) on H_2O_2 [37] takes place after the system reaches the transition state. This concerted proton transfer step, which has also been observed in previous work [65], ultimately leads to the sulfenic acid (Cys-SO$^-$) product, driven by the expulsion of a stable H_2O molecule (Equation 2).

$$\text{Cys-SH} + H_2O \rightleftharpoons \text{Cys-S}^- + H_3O^+ \qquad \text{Equation 1}$$

Cys-S$^-$ + H_2O_2 ⇌ Cys-SO$^-$ + H_2O Equation 2

Selected internal parameters of oxidation of cysteines by hydrogen peroxide are depicted in Supplementary Tables 7, 8 and 9.

Coordinates of transition states for GLRX oxidation by H_2O_2

Residues 22-23-24

final geometry:

angstroms

atom	x	y	z
O1	3.4186674498	-12.0189836655	5.2297444765
H2	3.3572874655	-12.9586461927	5.4676811613
O3	2.3636830136	-12.3180793871	3.6110147706
H4	2.1460116169	-11.3971691566	3.8548457538
C5	5.3562459972	-11.0427045684	-4.2884725118
C6	5.0021229430	-12.5689729030	-4.1217640671
O7	4.1068892852	-13.0308996205	-4.8210403493
N8	5.6292417902	-13.3812342105	-3.1856609490
C9	5.2525772481	-14.7508298979	-2.9389211996
C10	4.0790316913	-14.8953226560	-1.9724593471
O11	3.4368729605	-15.9503059972	-1.9499868767
C12	6.5106235211	-15.3955977873	-2.3851045785
O13	7.0648151329	-14.6626870893	-1.2746904006
C14	7.6260669711	-15.5351140684	-3.4184947280
H15	6.3580196017	-12.9984312390	-2.5954480023
H16	4.9852995365	-15.2286245883	-3.8834863131
H17	6.2405900747	-16.3998035827	-2.0373988974
H18	6.4394513589	-14.6843427792	-0.5303545183
H19	8.4610010297	-16.0824864024	-2.9712282719
H20	7.2742233459	-16.0944619163	-4.2912899174
H21	7.9935036856	-14.5618714910	-3.7622408477
N22	3.8751827492	-13.8783762063	-1.1000783809
C23	2.7825834634	-13.9294367306	-0.1321338351
C24	1.4475282933	-14.1135597827	-0.8729858229
O25	1.1810833932	-13.5000258016	-1.9150606468
C26	2.7885498176	-12.6489579955	0.7364039643
S27	1.2986162112	-12.3922306017	1.7678040908
H28	4.2548970864	-12.9584253778	-1.3377668205
H29	2.9641108592	-14.7935925157	0.5061793703
H30	2.9285861434	-11.7899815074	0.0801802600
H31	3.6397288474	-12.6896760290	1.4248981295
N32	0.5751320336	-14.9866331275	-0.3064029335
C33	-0.7373583863	-15.2388194797	-0.8753560417
C34	-1.7666754934	-14.1035106789	-0.7575634860
O35	-2.6596497387	-13.9872508110	-1.5953128867
C36	-1.2554330025	-16.4799273860	-0.1391616626

C37	-0.5214645358	-16.4366513056	1.2104969169
C38	0.8498801670	-15.8617227163	0.8594717191
H39	-0.6402602658	-15.4293193280	-1.9556677588
H40	-0.9489731360	-17.3738268339	-0.6936286211
H41	-2.3437034089	-16.4980576785	-0.0361614835
H42	-0.4559286641	-17.4177633773	1.6878066204
H43	-1.0275664954	-15.7606066607	1.9021752820
H44	1.2533659160	-15.2688315466	1.6824766532
H45	1.5647663598	-16.6421688611	0.5699569086
N46	-1.6364277554	-13.2859592661	0.3294029155
C47	-2.5383891003	-12.1601042819	0.6163400314
H48	-0.8644979348	-13.3964723597	0.9861702028

Residues 7-8-9

final geometry:

	angstroms		
atom	x	y	z
C1	9.4860975888	5.1851918191	1.2715823576
N2	10.3650518203	6.1641804610	1.6026068172
C3	10.1769709993	7.6187968987	1.4717506547
C4	9.9416585154	8.0686919440	0.0338889869
O5	9.0406658096	8.8975297130	-0.1475354211
C6	11.4102305447	8.4513654519	1.9447333593
C7	11.7647023883	8.3870498574	3.4461760950
H8	11.1691646466	5.8731193514	2.1517273027
H9	9.3038275514	7.8989550792	2.0724225128
H10	11.2487167914	9.5094736427	1.7265550949
H11	12.2962265304	8.1664374040	1.3746700957
N12	10.6110051626	7.4248857342	-0.9604570569
C13	10.4048621930	7.8109945500	-2.3516889923
C14	9.4212394778	6.8990582792	-3.1020399345
O15	9.4386501603	6.8404889596	-4.3319809353
C16	11.7071345808	8.1044457207	-3.1168160002
S17	12.2217134492	9.8751960575	-3.0303987071
H18	11.4352587874	6.8809847261	-0.7022615483
H19	9.8545371574	8.7597138845	-2.3356329710
H20	12.5190106849	7.4710570662	-2.7307053494
H21	11.5374866649	7.8300353545	-4.1600037337
N22	8.5072984936	6.2642865479	-2.3190961484
C23	7.2975527308	5.7526157105	-2.9134506117
C24	6.0104326659	6.3120819898	-2.2784565623
O25	4.9820782970	6.3043272569	-2.9520341137
C26	7.2811344067	4.2148269855	-2.9333755700
C27	8.3154882101	3.6060120577	-3.8934383992
H28	8.6701299745	6.2241903883	-1.3148402959
H29	7.2007085280	6.1215924468	-3.9396127499
H30	6.2920711098	3.8558542415	-3.2210101121
H31	7.4598231108	3.8355717983	-1.9244087467
N32	6.0339185551	6.7875460541	-1.0283716862
O33	8.1544122888	10.8447110021	-2.6414980980

H34	8.1331716333	10.2034899879	-1.9051732327
O35	9.7489878500	10.5405564854	-3.1328801620
H36	10.2140325215	11.0957700042	-2.4803447732

Residues 82-83-84

final geometry:

	angstroms		
atom	x	y	z
C1	-3.3647013154	-2.9132831741	6.0202327530
N2	-3.8059290125	-4.1733607454	6.1886806609
C3	-3.7810787824	-5.2935481828	5.2488970588
C4	-5.1581416813	-5.7210488104	4.7472326144
O5	-6.0295558166	-4.8625605658	4.6295628240
H6	-4.2914819205	-4.3383124547	7.0913012622
H7	-3.3076246776	-6.1446909789	5.7437227973
H8	-3.1658179760	-5.0483504183	4.3794387503
N9	-5.3817165201	-7.0228328719	4.4199811716
C10	-6.5741051019	-7.2854532327	3.6224036199
C11	-7.8949576536	-7.1670848753	4.3667553501
O12	-8.8913517143	-6.8043929544	3.7644733872
C13	-6.6289162017	-8.7051196180	3.0232755913
S14	-5.3001032443	-9.0859829006	1.8593662294
H15	-4.6402897154	-7.7413945248	4.4356934990
H16	-6.6345291854	-6.5569286816	2.8107170405
H17	-7.5902164243	-8.7765067622	2.5049579447
H18	-6.6475155151	-9.4397233602	3.8368449969
N19	-7.8694376320	-7.6707544010	5.6426526183
C20	-9.0997035345	-7.5387176095	6.3826262791
C21	-9.4157584376	-6.0885018011	6.8238498920
O22	-10.5896571285	-5.7379203326	6.9331917389
C23	-9.0613338209	-8.5111924448	7.5706321067
O24	-7.9858599526	-8.2151098566	8.4308053492
H25	-6.9728353855	-7.7460603634	6.1083407046
H26	-9.9124784834	-7.9009090795	5.7417200337
H27	-8.9920625094	-9.5490365714	7.2390068441
H28	-9.9878797997	-8.4304537110	8.1442268022
N29	-8.3622245099	-5.2737577398	6.9752483350
O30	-3.1193648964	-9.1878805758	5.3825865644
H31	-2.3667631825	-8.7061219928	5.0088927948
O32	-3.9645555850	-9.1783958518	3.7053659576
H33	-4.3334553384	-10.0292314597	3.9782853719

Residues 78-79-80

final geometry:

	angstroms		
atom	x	y	z
O1	-0.6600447612	-0.1735736612	7.0061390455
H2	-1.1366069632	0.3840826735	6.3787826831
O3	-1.1529014792	1.1161414201	8.2689788171

H4	-0.2066000500	1.2876465445	8.3017918588
C5	0.0959711505	8.2066706717	4.6968171789
N6	-1.2414166735	8.2130533488	4.8454143247
C7	-2.0291790179	7.4344037350	5.7849590957
C8	-2.1063803575	5.9755726463	5.3478375196
O9	-2.0323002279	5.6448056284	4.1589235025
C10	-3.5364720121	7.7351686257	5.7139873762
C11	-3.9726889318	9.1149134333	6.1380603592
H12	-1.7823693909	8.7093880753	4.1565048898
H13	-1.6413156626	7.5386929728	6.8047667083
H14	-4.1312670076	7.0745343711	6.3496199733
H15	-3.9196264647	7.6013291694	4.6982126242
N16	-2.2932253342	5.1319163394	6.3751298018
C17	-2.1293356828	3.7132920490	6.2142218710
C18	-3.4264668858	3.0656910393	6.7174961637
O19	-4.0333015995	3.5435008597	7.6531111552
C20	-0.8173545962	3.3568314920	6.9414009044
S21	-0.9804602836	3.4020921747	8.7576125642
H22	-2.3314313956	5.4783532860	7.3275870358
H23	-2.0122232479	3.5438649479	5.1418254964
H24	-0.0912766551	4.1047010912	6.6074392735
H25	-0.4616275535	2.3779160913	6.6177530588
N26	-3.8489759107	2.0317554092	5.9285099357
C27	-5.0758205608	1.3700089713	6.2966032999
C28	-4.7285808933	0.0832575961	7.0448662703
O29	-5.4994582233	-0.2704744662	7.9243786294
C30	-5.9414356140	1.0869414769	5.0533974239
C31	-6.1766851569	2.3581892490	4.2232215550
C32	-7.2802134332	0.3434039010	5.3032195391
H33	-3.2641067692	1.6932964501	5.1688995980
H34	-5.6908314957	1.9910317474	6.9587723623
H35	-5.3599124815	0.4139744441	4.4177896874
N36	-3.6455121322	-0.6212498850	6.6837765183

Residues 25-26-27

final geometry:

	angstroms		
atom	x	y	z
O1	4.1204287845	-12.3571781586	1.3581582911
H2	3.8729720706	-12.1057551423	2.2634711178
O3	3.0321984918	-10.8945984917	0.6844819662
H4	3.6982715821	-10.9351505375	-0.0364139602
C5	-1.7300574561	-14.0949355623	-0.7398384436
N6	-1.8773620461	-13.3431807456	0.3457605748
C7	-2.7263575256	-12.1783705275	0.6320940639
C8	-2.2928160118	-10.8983268617	-0.0741307760
O9	-3.1523689186	-10.0497038181	-0.3138016226
C10	-2.6239337812	-11.8366965314	2.1418968232
C11	-2.5367409601	-13.0544280018	3.0559538950

H12	-1.2286444213	-13.5657667319	1.0996565411
H13	-3.7621541420	-12.4211654428	0.3921984090
H14	-3.4916185035	-11.2478303046	2.4458172293
H15	-1.7575331035	-11.2117712562	2.3605245984
N16	-1.0114136169	-10.7873557569	-0.5059618255
C17	-0.5899663106	-9.7117530278	-1.3807442503
C18	-1.1467458715	-9.8526379823	-2.7968777086
O19	-1.1753704758	-8.8611406017	-3.5118608663
C20	0.9467638958	-9.6641994976	-1.5372674818
S21	1.8670750038	-9.2197506922	-0.0215912559
H22	-0.2911532234	-11.3021904209	0.0037725721
H23	-0.9479287103	-8.7484806914	-1.0171922159
H24	1.1129667486	-8.9423741455	-2.3307462168
H25	1.3086308819	-10.6064752985	-1.9622686120
N26	-1.4843267588	-11.1240498056	-3.1886326672
C27	-2.2118538395	-11.2250160503	-4.4292208747
C28	-3.6356998048	-10.5967721378	-4.3519714680
O29	-4.1053140029	-10.0293803978	-5.3243976416
C30	-2.2849808453	-12.7019290505	-4.8857190911
C31	-0.9194668056	-13.4453965905	-4.9731466957
H32	-1.5329951649	-11.8541295721	-2.4867452141
H33	-1.6338954867	-10.6922838932	-5.1922621339
H34	-2.7646524012	-12.7112864823	-5.8681033971
H35	-2.9584008285	-13.2639868475	-4.2359375217
N36	-4.2517390640	-10.6662484756	-3.1694095206

Coordinates of minimized reactant state for GLRX oxidation by H_2O_2

Residues 22-23-24

final geometry:

	angstroms		
atom	x	y	z
O1	2.9232931687	-14.0631923186	3.7318171665
H2	2.6242633975	-13.1445575640	3.5508368108
O3	1.6610504576	-14.7766302389	3.5329740245
H4	1.2225532120	-14.1428197540	2.8989370237
C5	5.3555214909	-11.0486945462	-4.2887239987
C6	4.9711278606	-12.5776471778	-4.1443357359
O7	4.0738673065	-13.0068616574	-4.8632350534
N8	5.5691523500	-13.4336654969	-3.2258409687
C9	5.1736628732	-14.8030388880	-3.0091522457
C10	3.9697820611	-14.9531934297	-2.0760478971
O11	3.2910415441	-15.9786918905	-2.1125736934
C12	6.3964929916	-15.4647908891	-2.3935433065
O13	6.8660679268	-14.7619499425	-1.2379436035
C14	7.5796854137	-15.5815288567	-3.3520945211
H15	6.3104185217	-13.0829453710	-2.6332264613
H16	4.9361834615	-15.2688677452	-3.9668494731
H17	6.0973375412	-16.4815878141	-2.1068414260
H18	6.1571723535	-14.7644343018	-0.5776286113

H19	8.3811866104	-16.1376950461	-2.8600626946
H20	7.2932003501	-16.1180852987	-4.2602796494
H21	7.9700708485	-14.6018259094	-3.6433679655
N22	3.8099307847	-13.9702840582	-1.1535522571
C23	2.7077952415	-14.0092745898	-0.1907943295
C24	1.3782315187	-14.1614573252	-0.9340680310
O25	1.1064858940	-13.5017867534	-1.9386967951
C26	2.7392369108	-12.7223927846	0.6666950691
S27	1.2477094467	-12.3445321142	1.6697116199
H28	4.2059512689	-13.0556265855	-1.3679574098
H29	2.8729657181	-14.8661607604	0.4601551395
H30	2.9086635403	-11.8820911731	-0.0053251078
H31	3.6104333305	-12.8024273736	1.3236980331
N32	0.4999091934	-15.0633287781	-0.4081263085
C33	-0.8192890716	-15.2428659694	-0.9757422596
C34	-1.8361522864	-14.1051306316	-0.7917779621
O35	-2.7763505210	-13.9938754562	-1.5782226920
C36	-1.3877511926	-16.5014671471	-0.2928980684
C37	-0.5903330952	-16.5910358091	1.0173692007
C38	0.7944925628	-16.0887021476	0.6183650507
H39	-0.7391238452	-15.3901645280	-2.0659492633
H40	-1.1659806911	-17.3748475391	-0.9152507485
H41	-2.4687591436	-16.4630430734	-0.1390660817
H42	-0.5692977044	-17.5999670749	1.4338372369
H43	-1.0136157133	-15.9238554348	1.7718247379
H44	1.3183023810	-15.6607351632	1.4734678779
H45	1.4106081185	-16.8815078705	0.1737932429
N46	-1.6476356370	-13.2885287589	0.2895315372
C47	-2.5368765599	-12.1607363375	0.6115334397
H48	-0.8056045808	-13.3576184284	0.8665425182

Residues 7-8-9

final geometry:

	angstroms		
atom	x	y	z
C1	9.4904928205	5.1851840846	1.2633355925
N2	10.3738355380	6.1637524153	1.5821858928
C3	10.1743222765	7.6186201562	1.4824929618
C4	9.9124758187	8.1380649571	0.0729407386
O5	9.0882878998	9.0516954962	-0.0261710537
C6	11.4127452366	8.4481382377	1.9481829605
C7	11.7657814650	8.3850606915	3.4475867501
H8	11.1845699124	5.8707729973	2.1201197560
H9	9.3132112089	7.8782769874	2.1091832495
H10	11.2611101553	9.5091858254	1.7356752356
H11	12.2945442458	8.1550237931	1.3760617112
N12	10.4954403380	7.5087416617	-0.9888180930
C13	10.2389532086	7.9970485581	-2.3530490572
C14	9.3118178761	7.0405018540	-3.1300323525
O15	9.3461587996	6.9747322769	-4.3483335023

C16	11.5268723200	8.3488863946	-3.1298976821
S17	11.3589923407	9.7383603221	-4.3365919328
H18	11.2775069708	6.8869647621	-0.7982809118
H19	9.6494064048	8.9121970526	-2.2189254423
H20	11.8850427168	7.4505027073	-3.6469950666
H21	12.2846826854	8.6200942286	-2.3878610991
N22	8.4666422980	6.3024896607	-2.3431228822
C23	7.2695186970	5.7543968087	-2.9305227824
C24	5.9904632027	6.3037760099	-2.2769321378
O25	4.9526719222	6.2871415466	-2.9357173498
C26	7.2794088901	4.2161503948	-2.9341766104
C27	8.3156195095	3.6076280920	-3.8933286436
H28	8.6311678192	6.2795224539	-1.3408402628
H29	7.1560626005	6.1089144229	-3.9597761668
H30	6.2943281661	3.8408276489	-3.2133997768
H31	7.4687319521	3.8505143859	-1.9222081706
N32	6.0307752113	6.7852837509	-1.0283165749
O33	8.5874372468	10.9992180844	-3.6489004857
H34	9.5747112566	10.8571027346	-3.5780097962
O35	8.3463091234	10.3390784519	-4.9374763300
H36	9.1999416340	9.8256732294	-5.0062816731

Residues 82-83-84

final geometry:

	angstroms		
atom	x	y	z
O1	-6.7854794615	-12.2864570606	3.6716190163
H2	-7.3567521000	-11.5167071634	3.4440628245
O3	-6.2020824958	-12.5337134501	2.3559921275
H4	-6.2310687860	-11.5919305527	2.0025472297
C5	-3.3275085686	-2.9096958505	6.0377013081
N6	-3.6864515317	-4.1817993856	6.2463984883
C7	-3.5865222849	-5.2876324536	5.3134715440
C8	-4.9491180715	-5.7023550965	4.7638678208
O9	-5.8232213382	-4.8904278438	4.5207854442
H10	-4.1876153486	-4.3489800879	7.1403727046
H11	-3.1257123531	-6.1318896086	5.8309715876
H12	-2.9411722337	-5.0313086540	4.4693807565
N13	-5.1082649912	-7.0500281217	4.5494448774
C14	-6.1846703326	-7.5104744679	3.6544954355
C15	-7.5860895724	-7.2963277948	4.2634221141
O16	-8.4985924387	-6.8665079675	3.5886109243
C17	-5.8970595895	-8.9808056321	3.2821711577
S18	-6.9385328476	-9.6144763803	1.9195919592
H19	-4.2869649633	-7.6376438964	4.6305666264
H20	-6.1834085039	-6.8994299236	2.7481730285
H21	-6.0013186219	-9.6058207729	4.1790779498
H22	-4.8314100108	-9.0153138021	3.0003452478
N23	-7.6888512828	-7.6728901990	5.5805294298
C24	-8.9769731515	-7.5384946267	6.2139557322

C25	-9.3693017708	-6.0983497373	6.6551927179
O26	-10.5543881278	-5.7689769920	6.6820205941
C27	-9.0053054833	-8.5190677368	7.3965544752
O28	-8.0051615589	-8.1903929588	8.3364693293
H29	-6.8514077895	-7.8218461782	6.1234092011
H30	-9.7284049291	-7.9095260600	5.5066184393
H31	-8.8823862256	-9.5535232252	7.0683659252
H32	-9.9755583366	-8.4673172577	7.8966226972
N33	-8.3538058921	-5.2689115421	6.9296052900

Residues 78-79-80

final geometry:

	angstroms		
atom	x	y	z
O1	-1.1149455360	0.3969666315	8.3881250771
H2	-0.7101639993	1.3055121231	8.5172368377
O3	-1.9929186234	0.3965642978	9.5557444114
H4	-2.2447491785	1.3504122012	9.5510979047
C5	0.0947571622	8.2053420059	4.6979533383
N6	-1.2425526668	8.2107678860	4.8576010909
C7	-2.0025687973	7.4198747675	5.8114389061
C8	-2.0452324203	5.9634275093	5.3618111564
O9	-2.1303282813	5.6660631371	4.1614022799
C10	-3.5243347127	7.6982801546	5.7995602175
C11	-3.9765182847	9.0998820316	6.1587898360
H12	-1.7972789810	8.7117028938	4.1835110606
H13	-1.5861445784	7.5285200336	6.8195999205
H14	-4.0474103622	7.0480583091	6.5052158193
H15	-3.9656024711	7.4949446119	4.8199435698
N16	-2.0077073171	5.0837081161	6.3690704180
C17	-2.1253859130	3.6811161648	6.1298808963
C18	-3.5248460464	3.2180657686	6.5484031806
O19	-4.2382397134	3.8780532259	7.2758977841
C20	-0.9953808372	2.9517285591	6.8601134863
S21	-0.9919627880	3.3351599594	8.6603351797
H22	-1.9335421725	5.3761875050	7.3393001763
H23	-2.0262281615	3.5446546403	5.0510464580
H24	-0.0588561567	3.2748337048	6.3946288733
H25	-1.1094200868	1.8774165057	6.6800006343
N26	-3.8559319046	2.0383878518	5.9472788544
C27	-5.0850832095	1.3759905437	6.3063975334
C28	-4.7278406885	0.0851139207	7.0443745224
O29	-5.5043314886	-0.2804612057	7.9146010187
C30	-5.9438370663	1.0913909303	5.0584400168
C31	-6.1778154574	2.3593408183	4.2233362975
C32	-7.2806967909	0.3429529331	5.3029961862
H33	-3.2089597648	1.6237291872	5.2830495329
H34	-5.6814046587	1.9852489568	6.9955942807
H35	-5.3540398141	0.4226348506	4.4256310682
N36	-3.6399172226	-0.6179936766	6.6853790417

Residues 25-26-27

final geometry:

atom	x	y	z
O1	4.1119886493	-12.4811521547	1.1690467835
H2	3.8518245388	-11.6355853025	0.7401399942
O3	4.2461226263	-12.1074065060	2.5614768524
H4	3.3259397453	-12.1682248131	2.8680884991
C5	-1.7318773409	-14.0933552021	-0.7408630630
N6	-1.8880209871	-13.3447820408	0.3443801715
C7	-2.7253608566	-12.1709871882	0.6285652972
C8	-2.2856179406	-10.8977628944	-0.0861024946
O9	-3.1539153826	-10.0579202069	-0.3416746102
C10	-2.6194821780	-11.8345500992	2.1394901104
C11	-2.5362245164	-13.0540243276	3.0552370557
H12	-1.2197401135	-13.5452463107	1.0863957067
H13	-3.7627244939	-12.4067204815	0.3901955015
H14	-3.4798065554	-11.2358696922	2.4441115085
H15	-1.7474151881	-11.2157361022	2.3530646261
N16	-1.0058520411	-10.7878317133	-0.5132806741
C17	-0.5687266674	-9.7170349815	-1.3808698534
C18	-1.1308602875	-9.8348329726	-2.7890472323
O19	-1.1496616652	-8.8500566267	-3.5076571751
C20	0.9781547932	-9.7566866790	-1.5248933649
S21	1.8919477045	-9.6884351628	0.0578449141
H22	-0.2463119761	-11.1852785712	0.0594274499
H23	-0.8765876964	-8.7420714913	-1.0056254434
H24	1.1859727246	-8.9186782485	-2.1752216799
H25	1.2424189670	-10.6339173843	-2.1323692129
N26	-1.4756676180	-11.1132133715	-3.1848382718
C27	-2.2062060193	-11.2204889045	-4.4231252293
C28	-3.6264093769	-10.5861459971	-4.3462762663
O29	-4.0859226802	-10.0038614077	-5.3159143802
C30	-2.2833500093	-12.6978116450	-4.8807751741
C31	-0.9193411528	-13.4445303465	-4.9734082352
H32	-1.5276501345	-11.8303381261	-2.4716732069
H33	-1.6264539861	-10.6893227056	-5.1850854640
H34	-2.7678226340	-12.7071683981	-5.8605428820
H35	-2.9544087907	-13.2578497207	-4.2267637896
N36	-4.2519764294	-10.6660289406	-3.1701423966

(angstroms)

Coordinates of minimized product state for GLRX oxidation by H_2O_2

Residues 22-23-24

final geometry:

atom	x	y	z
C1	5.3549836140	-11.0501047823	-4.2874880155

C2	4.9539910402	-12.5774405293	-4.1386330619
O3	4.0569306506	-12.9999822993	-4.8621192782
N4	5.5320640258	-13.4353967891	-3.2081458017
C5	5.1260717569	-14.8015793278	-2.9907417212
C6	3.9348538375	-14.9432165482	-2.0389867303
O7	3.2174298208	-15.9403928143	-2.0982587533
C8	6.3506579364	-15.4763621198	-2.3909445176
O9	6.8376691102	-14.7855031167	-1.2360481247
C10	7.5208994493	-15.5969244895	-3.3643357705
H11	6.2678981840	-13.0878603955	-2.6065586588
H12	4.8696877523	-15.2626969765	-3.9455046254
H13	6.0463593332	-16.4924419125	-2.1066646923
H14	6.1356579246	-14.7903111999	-0.5677287259
H15	8.3220842418	-16.1675570473	-2.8887002507
H16	7.2164847711	-16.1189577568	-4.2752327994
H17	7.9184133014	-14.6179874478	-3.6484925758
N18	3.8397858043	-13.9888214606	-1.0763710683
C19	2.7525912624	-13.9917866355	-0.0897050653
C20	1.4122720092	-14.1425726847	-0.8234080200
O21	1.1334028854	-13.4494997417	-1.8081531774
C22	2.7879275343	-12.6553746283	0.6811324166
S23	1.4340177671	-12.3373538843	1.8819443588
H24	4.2532413058	-13.0817914781	-1.2908210885
H25	2.9168058680	-14.8084806277	0.6128577712
H26	2.7871377525	-11.8470763611	-0.0510091927
H27	3.7171851394	-12.5882826605	1.2563969359
O28	1.9635879957	-12.9003605476	3.2951535968
N29	0.5320659527	-15.0495493213	-0.3215722747
C30	-0.7757607124	-15.2373419672	-0.9237428151
C31	-1.7846729013	-14.0871699966	-0.7771294928
O32	-2.6811065090	-13.9410112332	-1.6072531204
C33	-1.3646616806	-16.4862846014	-0.2386107246
C34	-0.5643732901	-16.5898528515	1.0698759525
C35	0.8264632198	-16.1091114661	0.6704539710
H36	-0.6686061788	-15.3977687471	-2.0099604094
H37	-1.1579016831	-17.3641834932	-0.8598520267
H38	-2.4455158239	-16.4296408619	-0.0855654691
H39	-0.5612483107	-17.6001828060	1.4847260301
H40	-0.9759974734	-15.9169899362	1.8268837517
H41	1.3844412715	-15.7242419084	1.5373976410
H42	1.4093044430	-16.9035744166	0.1836451553
N43	-1.6353200000	-13.2883703002	0.3234766278
C44	-2.5356873521	-12.1593662576	0.6167284733
H45	-0.8691121987	-13.4323347578	0.9828944648
O46	2.4532318597	-15.3612592687	3.1082075488
H47	2.2450403154	-14.3417849067	3.2156746562
H48	1.9532636964	-15.7752513059	3.8233537905

Residues 78-79-80

final geometry:

angstroms

atom	x	y	z
O1	-0.5909863194	0.0262390144	6.8777503774
H2	-1.0246679658	0.3448855822	7.7934596558
H3	-1.1799971333	0.4242357998	6.2282794981
C4	0.0891465575	8.1886594500	4.6918535612
N5	-1.2507212104	8.1582092932	4.8510828213
C6	-2.0079704471	7.3982557784	5.8452475054
C7	-2.0364325530	5.9081977215	5.5225375550
O8	-2.0460741666	5.5195594106	4.3501725896
C9	-3.5271412024	7.6886055140	5.8051627309
C10	-3.9766296428	9.0995268160	6.1579573699
H11	-1.8120641564	8.6456072115	4.1717671665
H12	-1.6007567194	7.5825983864	6.8459300088
H13	-4.0775478495	7.0482627420	6.4984299189
H14	-3.9377143953	7.4803484528	4.8137172190
N15	-2.0497886705	5.0971022213	6.6026083588
C16	-2.0687001496	3.6558913999	6.4751251984
C17	-3.5020978475	3.1717446234	6.7440451793
O18	-4.2182724163	3.7810536696	7.5131250231
C19	-0.9874856204	3.0503824885	7.4218633977
S20	-1.4612009682	2.4700485182	9.1058737994
H21	-2.2303266510	5.4958708831	7.5153547981
H22	-0.2234754176	3.8282783455	7.5288440294
O23	-1.7054184352	0.8598192071	8.9397294184
H24	-0.5120396343	2.1919083003	6.9430142970
H25	-1.7963499340	3.4578893860	5.4338156105
N26	-3.8640282018	2.0902298495	5.9900565290
C27	-5.0815204188	1.3906712561	6.3169813863
C28	-4.7302703069	0.0895820101	7.0455382621
O29	-5.5167101072	-0.2849920460	7.9029544287
C30	-5.9383106268	1.0984787432	5.0647662753
C31	-6.1770406564	2.3604597489	4.2238672526
C32	-7.2782501770	0.3453813939	5.3030764221
H33	-3.2127352771	1.7104930660	5.3097292545
H34	-5.7057256118	1.9810216245	6.9980243425
H35	-5.3457796228	0.4291327242	4.4355114114
N36	-3.6468038712	-0.6167442913	6.6809717477

Residues 82-83-84

final geometry:

angstroms

atom	x	y	z
O1	-3.6091491290	-9.0963938208	4.6996806030
H2	-4.3833367192	-9.8364362254	4.3531422174
H3	-3.0697276042	-8.9795911123	3.9065550308
C4	-3.3762672319	-2.9189885746	6.0135246536
N5	-3.8391174331	-4.1803597190	6.1637004315
C6	-3.8028890982	-5.3225078068	5.2365659269
C7	-5.1504500673	-5.7920408204	4.6940297114

O8	-6.0168141757	-4.9225967605	4.5607619067
H9	-4.3236029591	-4.3440604761	7.0667812536
H10	-3.3315814273	-6.1610399566	5.7544025625
H11	-3.1636518218	-5.0857489614	4.3825508219
N12	-5.3736206219	-7.0899146493	4.3355468882
C13	-6.6052741837	-7.2736412381	3.5654277187
C14	-7.8895620695	-7.0620649831	4.3472239648
O15	-8.8778798497	-6.6343478993	3.7746355481
C16	-6.9511952705	-8.6644761648	2.9475926782
S17	-5.6905465852	-9.8146882979	2.4045416219
H18	-4.6445947088	-7.8538729118	4.4813232790
H19	-6.6190421584	-6.5221459699	2.7732867324
H20	-7.6358118223	-8.4524248388	2.1204290492
H21	-7.4990233911	-9.2657151925	3.6794889437
O22	-5.3486433465	-10.5662023995	3.8255817355
N23	-7.8597897628	-7.5987317015	5.6065695300
C24	-9.0876197721	-7.5203354943	6.3581258520
C25	-9.4138587140	-6.0853409035	6.8354686865
O26	-10.5891192968	-5.7420168358	6.9554483125
C27	-9.0356799247	-8.5282419049	7.5164444752
O28	-7.9936627488	-8.2167489528	8.4080952094
H29	-6.9659596354	-7.8080688235	6.0322890930
H30	-9.8923598874	-7.8752773578	5.7036684193
H31	-8.9102173255	-9.5514198934	7.1564400499
H32	-9.9755465019	-8.5053158833	8.0726693724
N33	-8.3599109639	-5.2707438234	6.9847917845

Residues 25-26-27

final geometry:

	angstroms		
atom	x	y	z
O1	3.9591290000	-12.2096940000	1.3251360000
H2	3.6391860000	-11.4323780000	0.8029350000
H3	3.7223200000	-11.9582410000	2.2265900000
C4	-1.7276330200	-14.0905460600	-0.7428729600
N5	-1.8752270200	-13.3357850600	0.3388080400
C6	-2.7278760200	-12.1737690600	0.6219760400
C7	-2.3285890000	-10.8962300000	-0.1085630000
O8	-3.1891400000	-10.0233280000	-0.2629710000
C9	-2.6143770200	-11.8353090600	2.1318430400
C10	-2.5343580200	-13.0535800600	3.0518230400
H11	-1.2004120200	-13.5282440600	1.0775030400
H12	-3.7628890200	-12.4319380600	0.3962240400
H13	-3.4695860200	-11.2312420600	2.4402080400
H14	-1.7368700200	-11.2214040600	2.3354440400
N15	-1.0747820000	-10.8099320000	-0.6410350000
C16	-0.6975920000	-9.6988320000	-1.4932140000
C17	-1.2247310000	-9.8170900000	-2.8999950000
O18	-1.2780620000	-8.8277550000	-3.6105100000
C19	0.8319000000	-9.6932870000	-1.6466070000

S20	1.4476660000	-9.3902860000	0.0562550000
H21	-0.2757550000	-11.0845380000	-0.0336490000
H22	1.0846710000	-8.9404690000	-2.3847820000
O23	2.9226140000	-9.8631690000	0.2567900000
H24	1.1874730000	-10.6433260000	-2.0562490000
H25	-1.0360080000	-8.7368080000	-1.0987360000
N26	-1.5000280000	-11.1091980000	-3.2862560000
C27	-2.2600947500	-11.2385139600	-4.5045026000
C28	-3.6867497500	-10.6304909600	-4.3858886000
O29	-4.1824237500	-10.0701429600	-5.3509236000
C30	-2.3048717500	-12.7270289600	-4.9105296000
C31	-0.9225857500	-13.4472419600	-4.9763416000
H32	-1.5223390000	-11.8175850000	-2.5601760000
H33	-1.7095457500	-10.7063669600	-5.2881396000
H34	-2.7926257500	-12.7733339600	-5.8876476000
H35	-2.9645407500	-13.2797689600	-4.2386856000
N36	-4.2627407500	-10.6802509600	-3.1792406000

Residues 7-8-9

final geometry:

atom	x (angstroms)	y	z
C1	9.4906751129	5.1846010932	1.2589253692
N2	10.3678120613	6.1670974024	1.5757558895
C3	10.1247853388	7.6178737477	1.5588145291
C4	9.6835837376	8.2101467157	0.2247309000
O5	8.7384568725	8.9970613637	0.2600801156
C6	11.3796516144	8.4540209073	1.9617567253
C7	11.7635545251	8.3851167590	3.4502043716
H8	11.1835744917	5.8659912106	2.1001426733
H9	9.3204940308	7.8106145121	2.2787707675
H10	11.2199631476	9.5144686166	1.7530457622
H11	12.2399240988	8.1549228395	1.3611194091
N12	10.2690211581	7.7500378469	-0.9206220620
C13	9.8360810526	8.2608364651	-2.2186339585
C14	9.1638708706	7.1734319380	-3.0656009235
O15	9.2258480585	7.1720582740	-4.2857647306
C16	10.8733878245	9.0798308870	-2.9956960208
S17	10.3034174003	10.8641280084	-3.0530588009
H18	11.1056319610	7.1820816433	-0.8274386405
H19	8.9958910115	8.9653774959	-2.0307445606
H20	10.9543236894	8.6739999740	-4.0069049193
H21	11.8569225023	9.0507564963	-2.5140538534
O22	8.6800283221	10.7182903217	-3.1478929346
N23	8.4606380206	6.2446314711	-2.3323381727
C24	7.2534017396	5.7300026806	-2.9292362742
C25	5.9797569177	6.2894121966	-2.2726406669
O26	4.9407436120	6.2751327666	-2.9283811420
C27	7.2637004430	4.1955815020	-2.9381935357
C28	8.3147280569	3.6061023480	-3.8932531947

H29	8.5983513581	6.2216977869	-1.3248772951
H30	7.1431898760	6.0725117653	-3.9625507751
H31	6.2822875905	3.8208922449	-3.2313798561
H32	7.4425949210	3.8224976174	-1.9269259791
N33	6.0280853410	6.7790239856	-1.0266691613
O34	8.1266475658	10.9419819306	-5.6868578473
H35	8.2744420888	10.8014771332	-4.6837632388
H36	9.0351343384	11.1384119111	-5.9482450365

Supplementary tables

Supplementary Table 1: Primers used for site directed mutagenesis of GLRX

Mutation	Primer Sequence	
C8S	Forward	5'-cccagactggatcttgctgttcacaaactcctgag-3'
	Reverse	5'-ctcaggagtttgtgaacagcaagatccagtctggg-3'
C23S	Forward	5'-ctgcagtaggggctggtgggcttgatg-3'
	Reverse	5'-catcaagcccaccagcccctactgcag-3'
Y25S	Forward	5'-gggtctttctgcagctggggcaggtgggct-3'
	Reverse	5'-agcccacctgccccagctgcagaaagaccc-3'
C26S	Forward	5'-ttgggtctttctgctgtaggggcaggtgg-3'
	Reverse	5'-ccacctgcccctacagcagaaagacccaa-3'
D47A	Forward	5'-tgttagtggctgtgatggccacaaactccagaaga-3'
	Reverse	5'-tcttctggagtttgtggccatcacagccactaaca-3'
C79S	Forward	5'-actgcatccgcctatgctgtctttacctatgaaga-3'
	Reverse	5'-tcttcataggtaaagacagcataggcggatgcagt-3'
C83S	Forward	5'-gagattagatcactgcttccgcctatgcagtcttt-3'
	Reverse	5'-aaagactgcataggcggaagcagtgatctaatctc-3'
C79S + C83S	Forward	5'-actgcttccgcctatgctgtctttacctatgaaga-3'
	Reverse	5'-tcttcataggtaaagacagcataggcggaagcagt-3'

Supplementary Table 2: GLRX mutant plasmids produced by site directed mutagenesis (SDM)

Starting Plasmid	SDM Primers Used*	Resulting GLRX Mutant Plasmid
WT	C8S	C8S
WT	C23S	C23S
WT	C26S	C26S
WT	D47A	D47A
WT	C79S	C79S
WT	D83S	C83S
C8S	Y25S	C8S + Y25S
C26S	D47A	C26S + D47A
C26S	C8S	C26S + C8S
C26S	C79S	C26S + C79S
C26S	C83S	C26S + C83S
C8S, C26S, C83S	C79S	C8S + C26S + C79S
C26S	C8S, C79S†, C83S	C8S + C26S + C83S
C8S, C26S, C83S	C79S + C83S	C8S + C26S + C79S + C83S

† C79S SDM primer was added to reaction but not expressed in the final plasmid sequence.

Supplementary Table 3: Summary of all-atom MD simulations.

System	Number of Atoms	Simulation Box (Å × Å × Å)	Simulation Length (ns)
GLRX wildtype (C23-C26 linkage)	19,858	62 × 56 × 66	400 × 2
GLRX wildtype (reduced)	19,860	62 × 56 × 66	400 × 2
GLRX C83S mutants	28,510	66 × 66 × 66	400 × 2
GLRX C8S mutants	22,609	62 × 57 × 66	400 × 2
GLRX C79S mutants	28,464	66 × 66 × 66	400 × 2
GLRX dimerization (aligned to PDBID: 3UIW)	50,208	80 × 80 × 80	200 × 2
GLRX dimerization (starting from three free monomers)	136,260	111 × 111 × 111	200 × 2
Total			4800

Supplementary Table 4: GSSG docking score (Glide XP precision) of the top pose for each GLRX cysteine.

Cysteine	C23	C8	C83	C79	C26
XP score of the top pose (kcal/mol)	-7.1	-6.7	-7.1	-6.6	-5.1

Supplementary Table 5: Thermodynamic parameters calculated with the model compound $[CH_3S\cdots HOOH]^-$ using the B3LYP/6-31G*/B3LYP/6-31G** level of theory.

	E_a (kcal/mol)	$\Delta G^{\#}$ (kcal/mol)	ΔH (kcal/mol)
$[CH_3S\cdots HOOH]^-$	21.6	22.3	-30.3

Supplementary Table 6: Selected geometric parameters of the model compound $[CH_3S\cdots HOOH]^-$ in the reactant, transition, and product states.

	Reactant State	Transition State	Product State
S-O-O angle (Degree)	77.0	167.7	96.4
S-O1 distance (Å)	3.1	2.2	1.6
O1-O2 distance (Å)	1.5	1.9	2.6

Supplementary Table 7: Selected geometric parameters of the thiolate oxidation of cysteines in the reactant state.

Reactant State	C8	C23	C79	C83	C26
S-O-O angle (Degree)	76.7	73.7	87.2	82.5	61.6
S-O1 distance (Å)	3.1	3.1	3.0	3.0	3.7
O1-O2 distance (Å)	1.5	1.5	1.5	1.5	1.5

Supplementary Table 8: Selected geometric parameters of the thiolate oxidation of cysteines in the product state.

Product State	C8	C23	C79	C83	C26
S-O-O angle (Degree)	105.1	109.9	110.3	100.5	131.1
S-O1 distance (Å)	1.6	1.6	1.6	1.6	1.6
O1-O2 distance (Å)	2.6	2.5	2.5	2.4	2.8

Supplementary Table 9: Selected internal parameters of the thiolate oxidation of cysteines in the transition state.

Transition State	C8	C23	C79	C83	C26
Imaginary frequency (cm^{-1})	-377	-360	-339	-156	-376
S-O-O angle (Degree)	160.4	172.5	141.7	170.6	177.9
H1-O2 distance (Å)	2.1	2.0	2.0	2.0	2.2

Supplementary Figure Legends

Supplementary Figure 1. Validation of cysteine to serine mutations on single mutants. Mass spectra confirming mutations were valid on single GLRX cysteine – serine mutants.

Supplementary Figure 2. Validation of cysteine to serine mutations on multiple mutants. Mass spectra confirming mutations were valid on multiple GLRX cysteine – serine mutants.

Supplementary Figure 3. Validation of protein S glutathionylation on GLRX peptides. MSMS spectra of the cysteine containing peptides that were identified and quantified shown in Figure 4. The coverage of measured fragment ions is included for VVVFIKPTCPYCR to show the y2 C(GSH/O3), y3 and y4 ions used for PRM quantification.

Supplementary Figure 4. Free energy profile of the reduction of H_2O_2. As performed with the model compound $[CH_3S^{\cdots}HOOH]^-$ using B3LYP/6-31G* level of theory.

Supplementary Figure 5. Time evolution of protein Ca RMSD in our MD simulations. These data show that mutation of cysteines to serine does not change the tertiary structure of GLRX. RMSD of 400 ns MD simulation of i. GLRX wildtype with C23-C26 disulfide bond (black circle) and reduced WT GLRX (orange square). ii. GLRX C8S mutant, iii. GLRX C83S mutant, and iv. GLRX C79S mutant.

Supplementary Figure 6. Comparison of pK_a of cysteines calculated with the PROPKA program. It is shown that the most acidic cysteine is C23 and C26 is the least acidic one; the remaining cysteines are in between.

Supplementary Figure 7. Reduction of WT GLRX by GSSG is reversed by DTT. Reduction of WT GLRX activity induced by incubation with 50 mM GSSG is reversed following incubation with 100 mM DTT for 10 min. Mean ± SD, n = 3. Statistics performed one-way ANOVA with Tukey correction, * $p \leq 0.05$, ** $p \leq 0.01$, *** $p \leq 0.001$, **** $p \leq 0.0001$.

Supplementary Figure 8. Snapshot of our GLRX dimer after 196 ns simulation starting from a model aligned to the zebrafish Grx2 protein (PDBID: 3UIW). The protein-protein interface is shown in surface and the C8 residues are in spheres. The distance of S groups is 8.9 Å.

Supplementary Figure 9. The top docking poses corresponding to the lowest distances of the sulfur atoms on cysteines (shown in yellow spheres) and GSSG (green sticks). The residues participating in hydrogen bonding with GSSG are shown in magenta.

Supplementary Figure 10. Illustration of the model compound $[CH_3S^{\cdots}HOOH]^-$ in the reactant, transition and product states.

Supplementary Figure 11. Validation of GLRX antibody. Antibody was validated using lung tissues from WT or *Glrx*$^{-/-}$ mice (n=4 per group). Although some non-specific reactivity is present, the GLRX immunoreactivity at 12 kDa is absent in the *Glrx*$^{-/-}$ mice.

Supplementary Figure 1

A. **C8S Raw file: QE+_077**

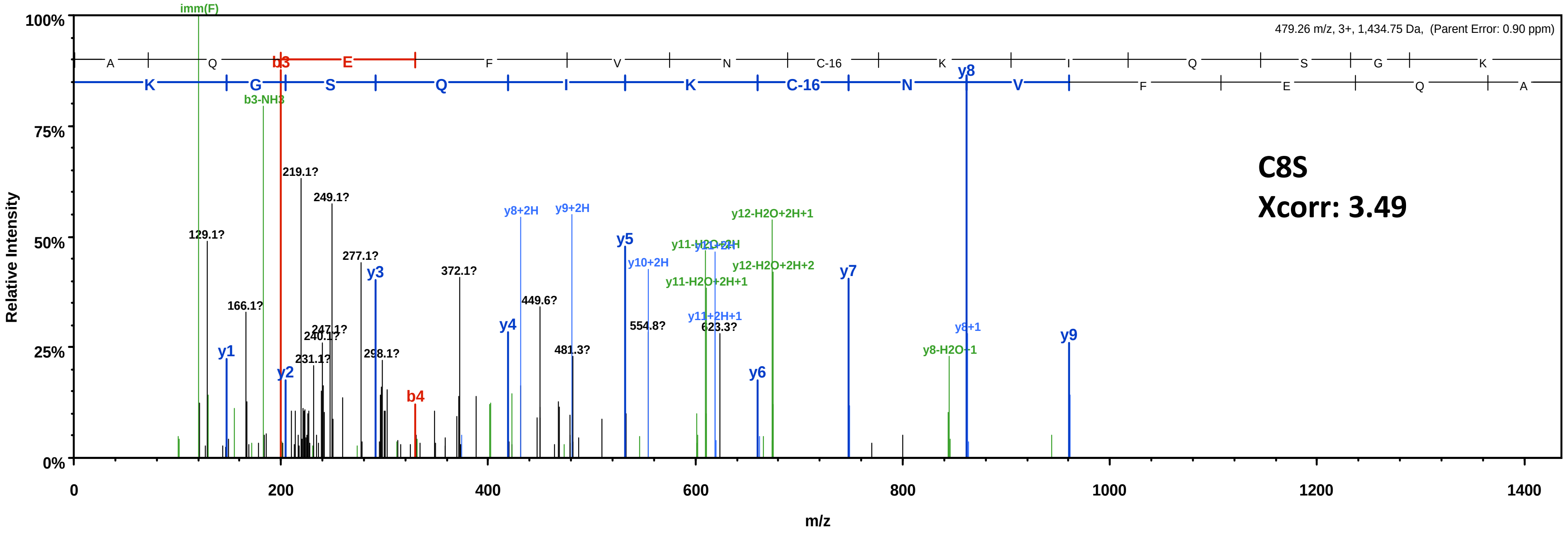

B. **C23S Raw file: QE+_078**

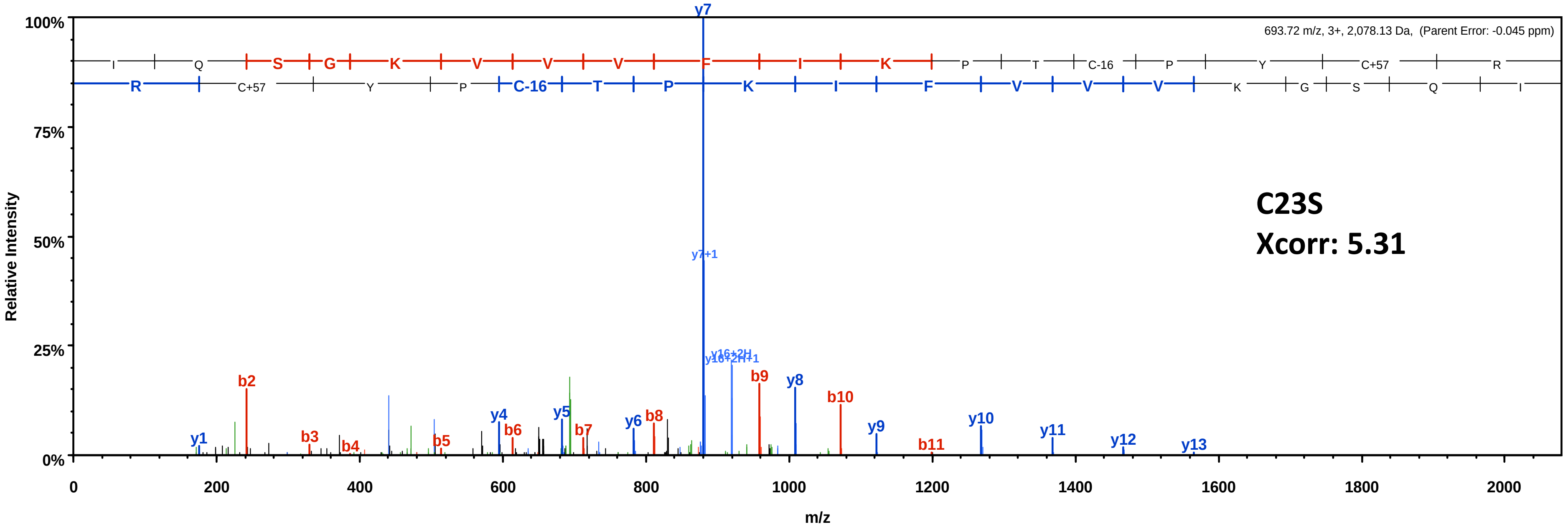

C. **C26S Raw file: QE+_080**

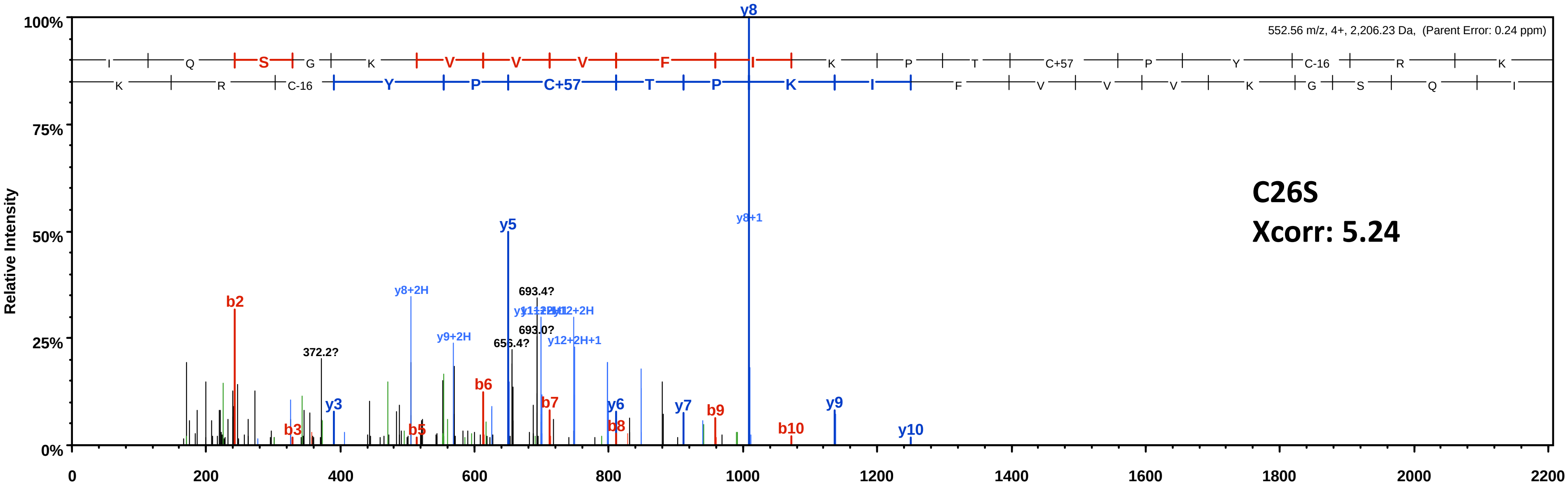

Supplementary Figure 1

D.

C79S Raw file: QE+_081

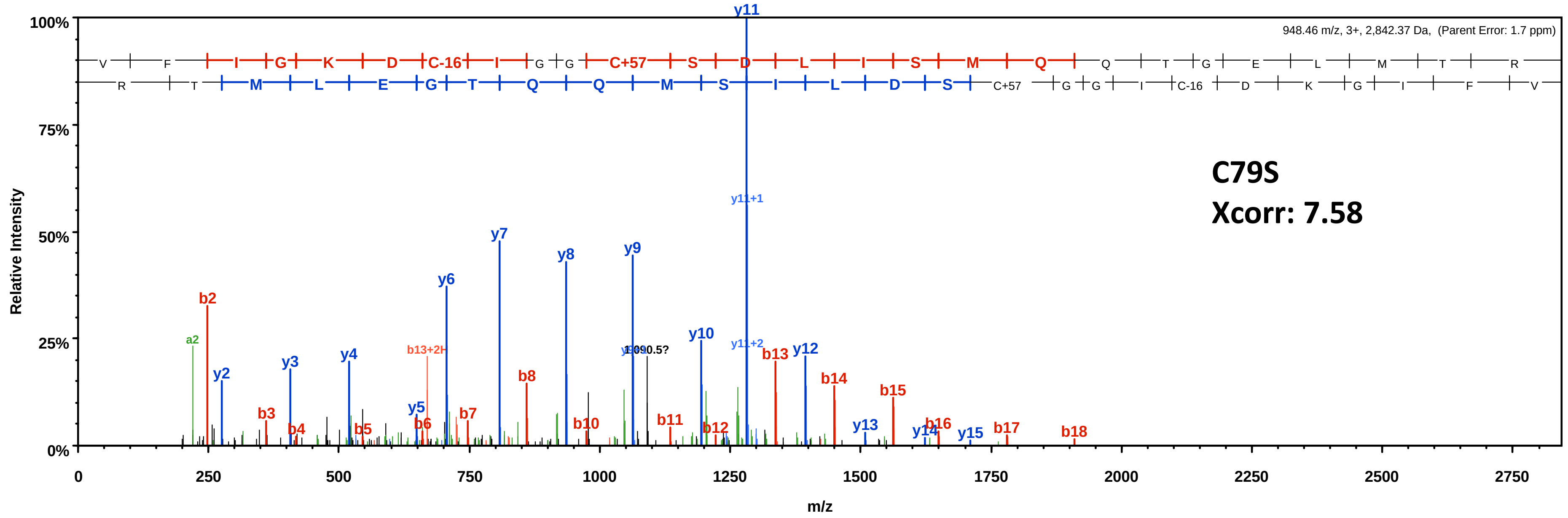

E.

C83S Raw file: QE+_082

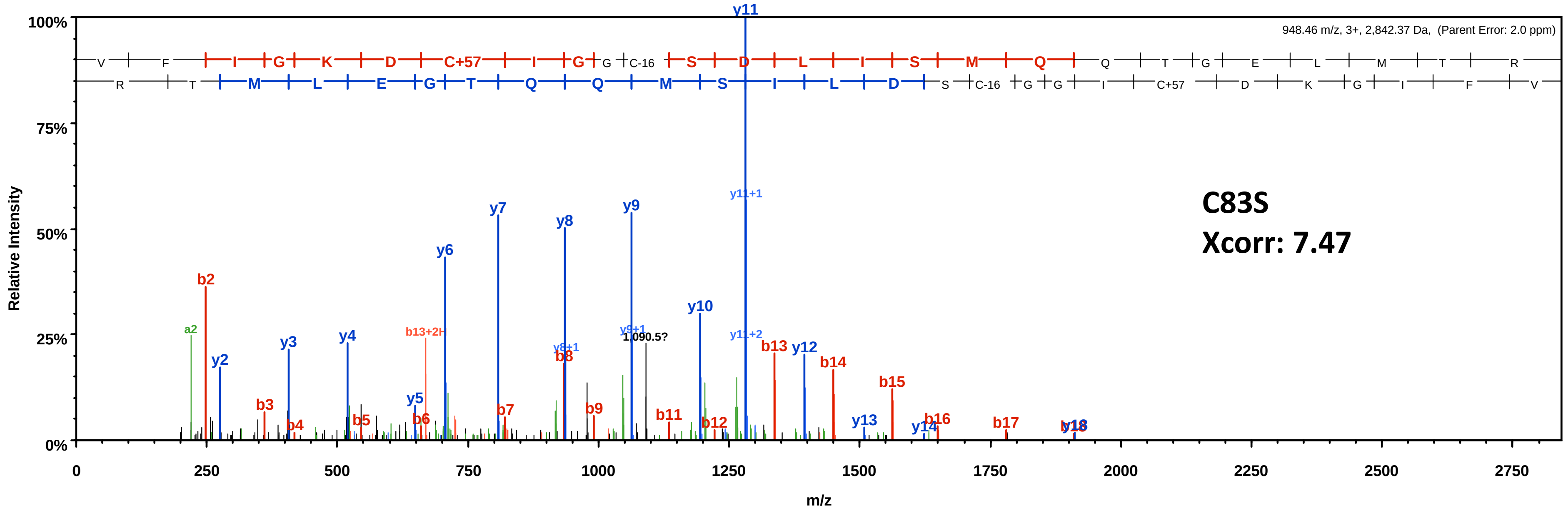

Supplementary Figure 2

A.

C8S/C26S Raw file: QE+_0505

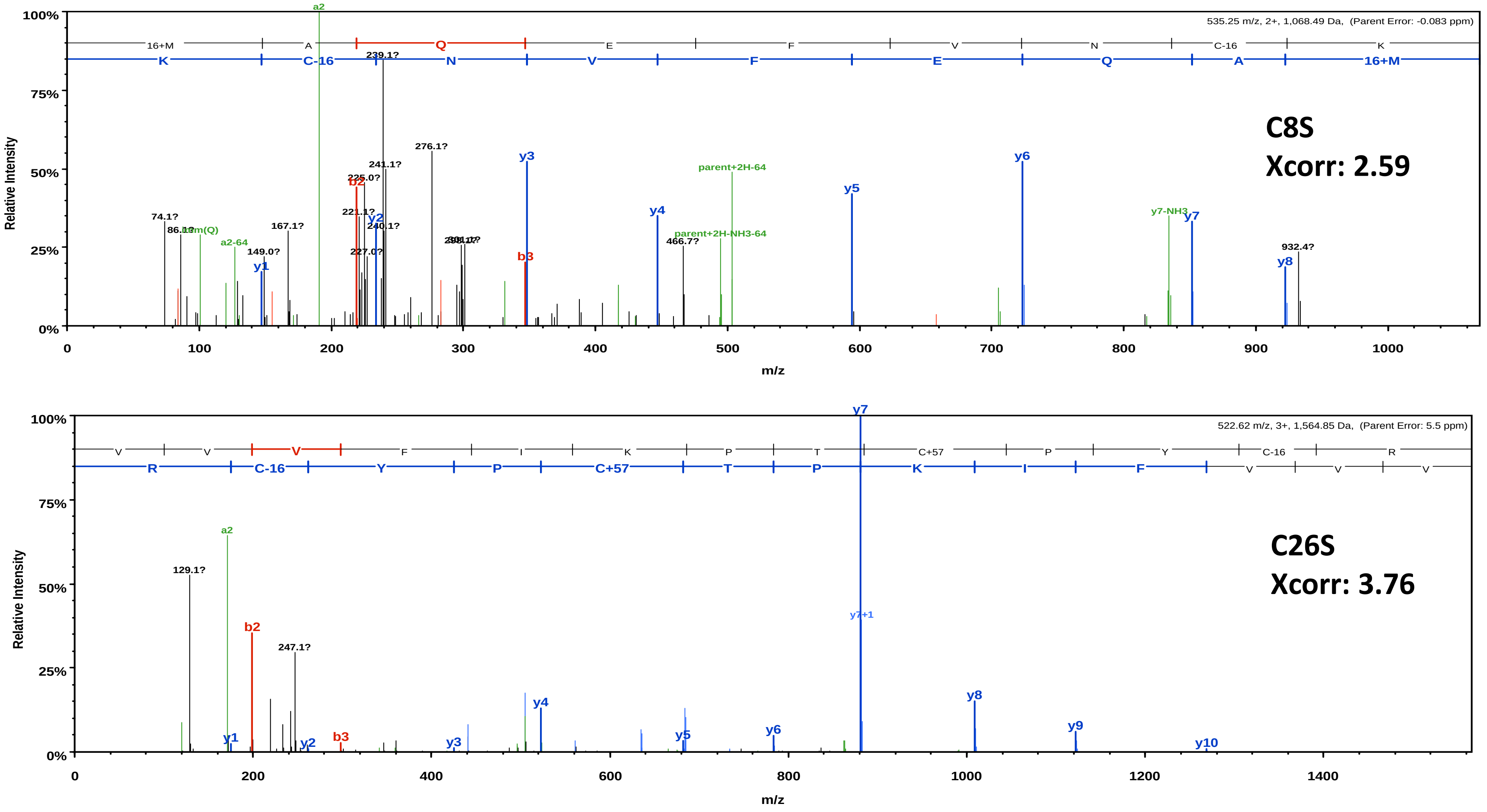

B.

C26S/C79S Raw file: QE+_0471

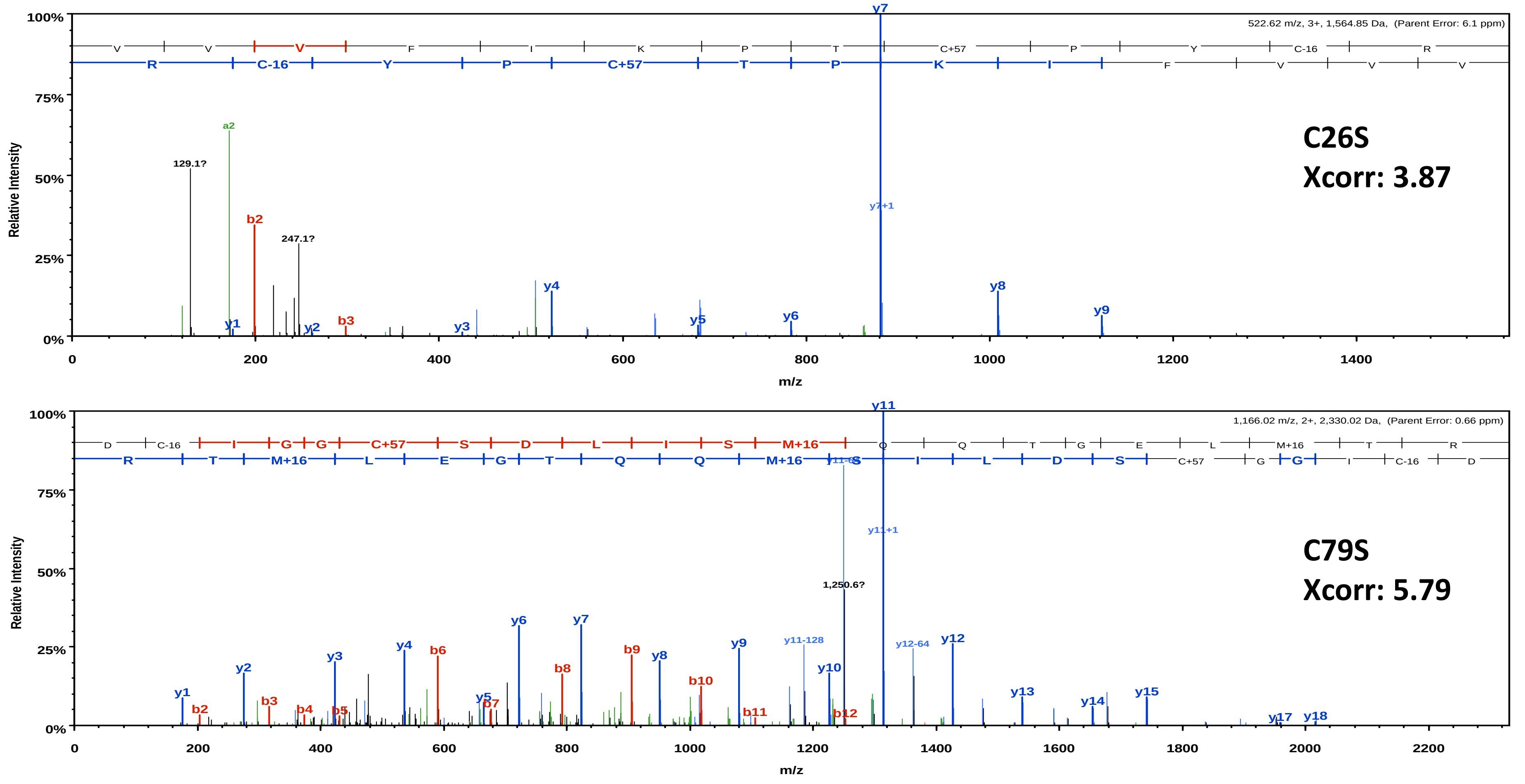

Supplementary Figure 2

C.

C26S/C83S Raw file: QE+_0474

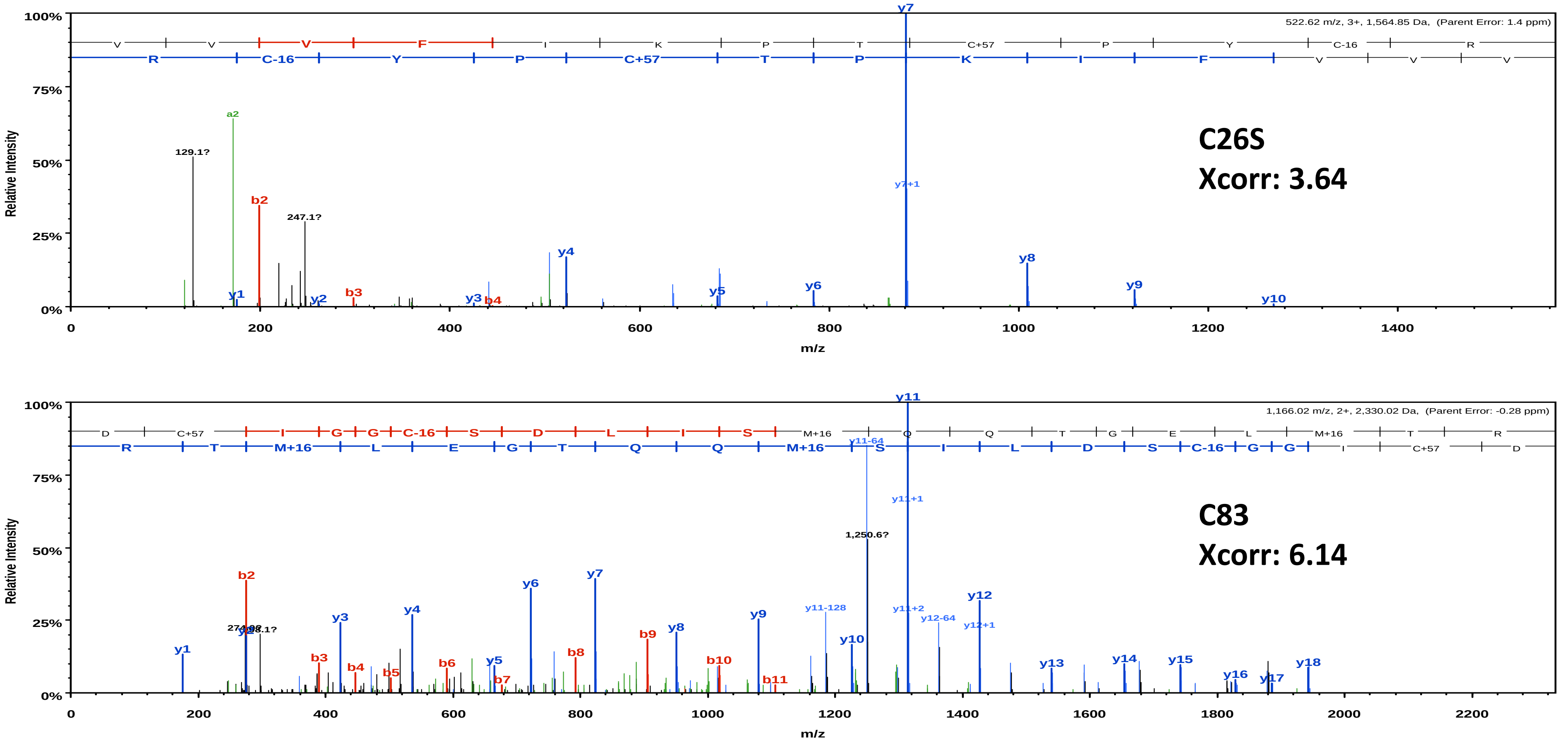

Supplementary Figure 2

D. C8S/C26S/C79S Raw file: QE+ _0480

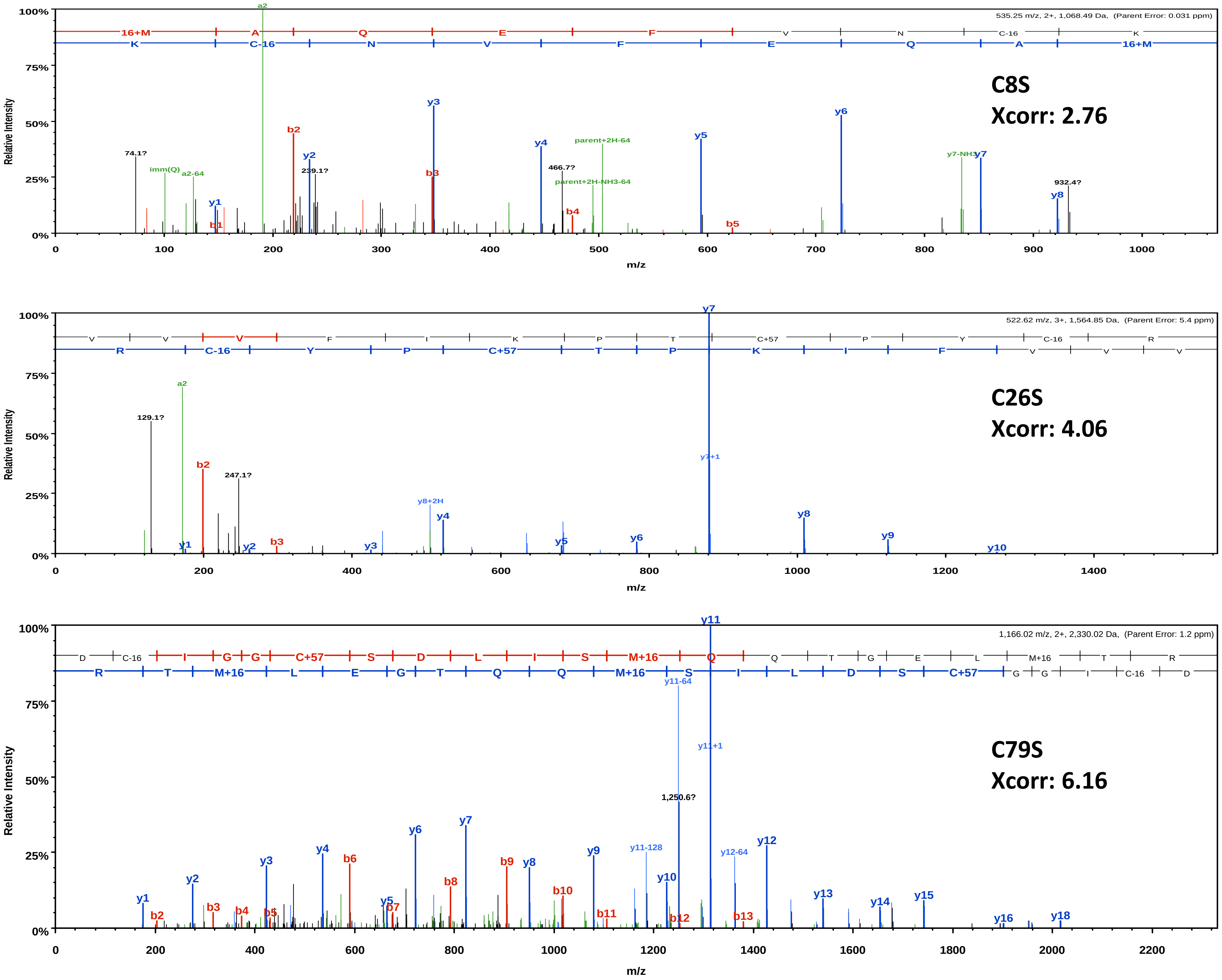

Supplementary Figure 2

E. **C8S/C26S/C83S** **Raw file: QE+_0477**

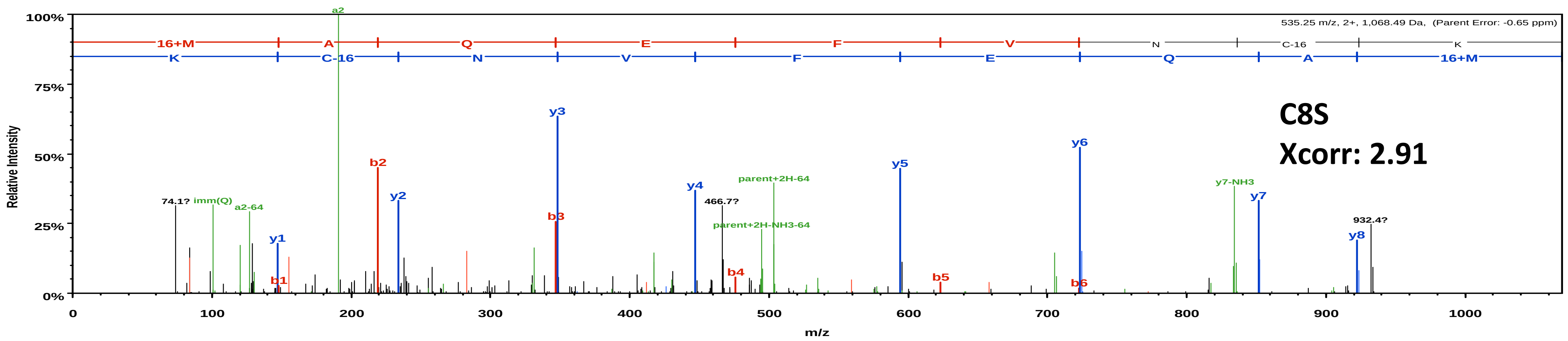

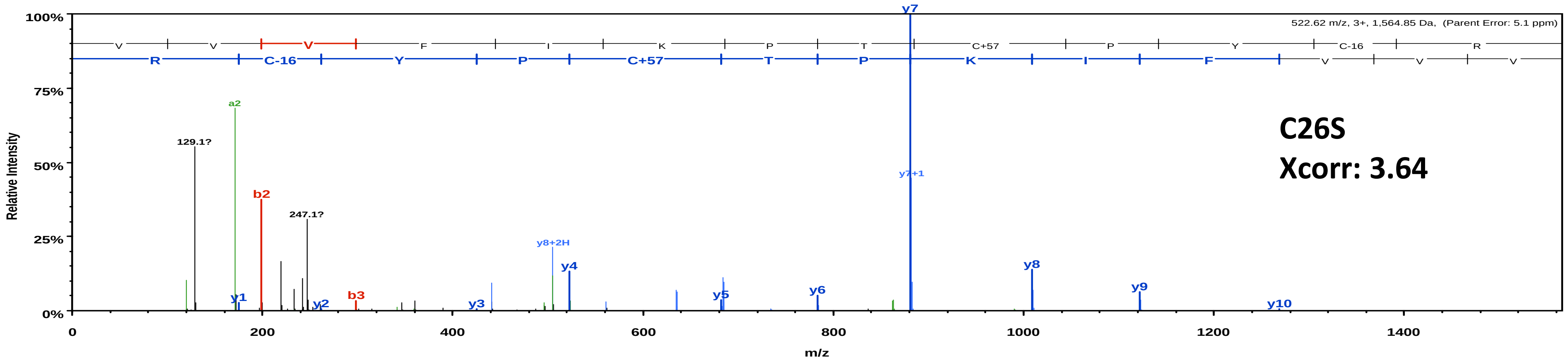

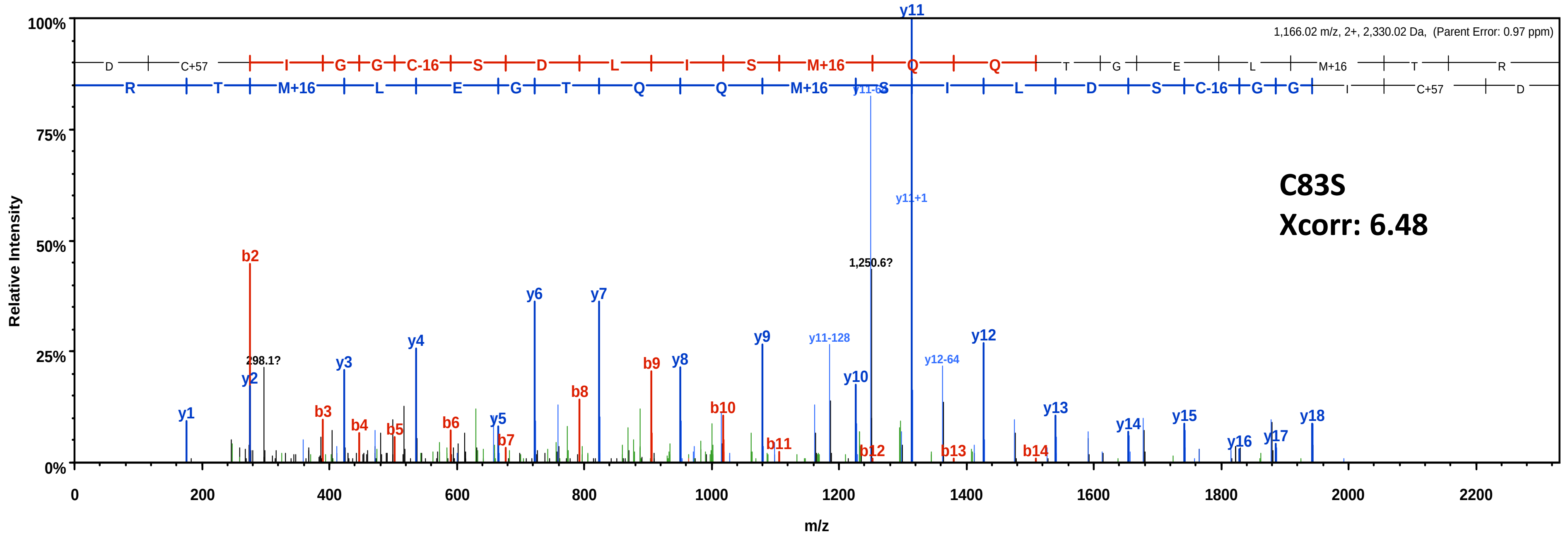

Supplementary Figure 2

F. C8S/C26S/C79S/C83S   Raw file: QE+_0465

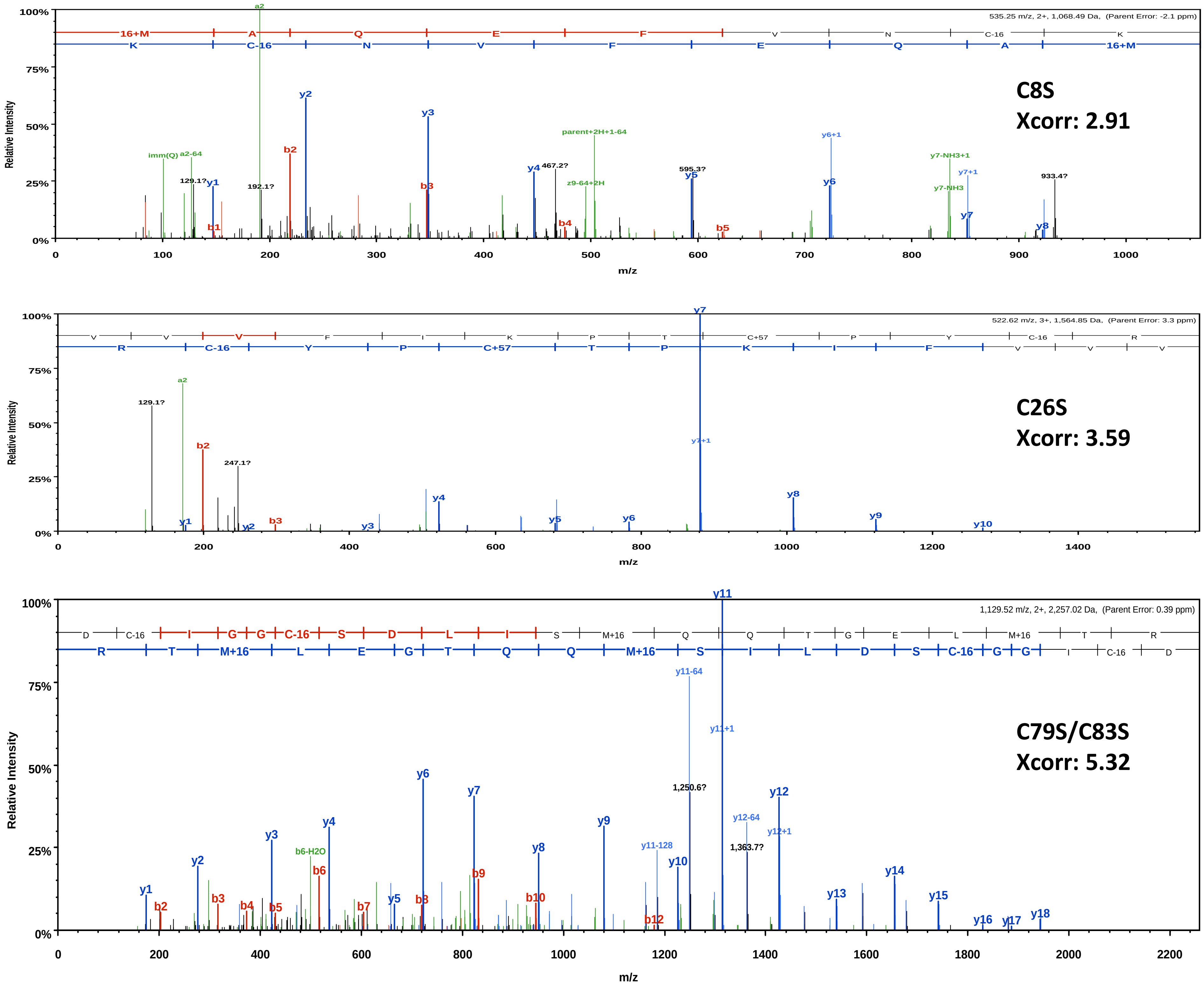

Supplementary Figure 3

M(O)AQEFVNC(CAM)K

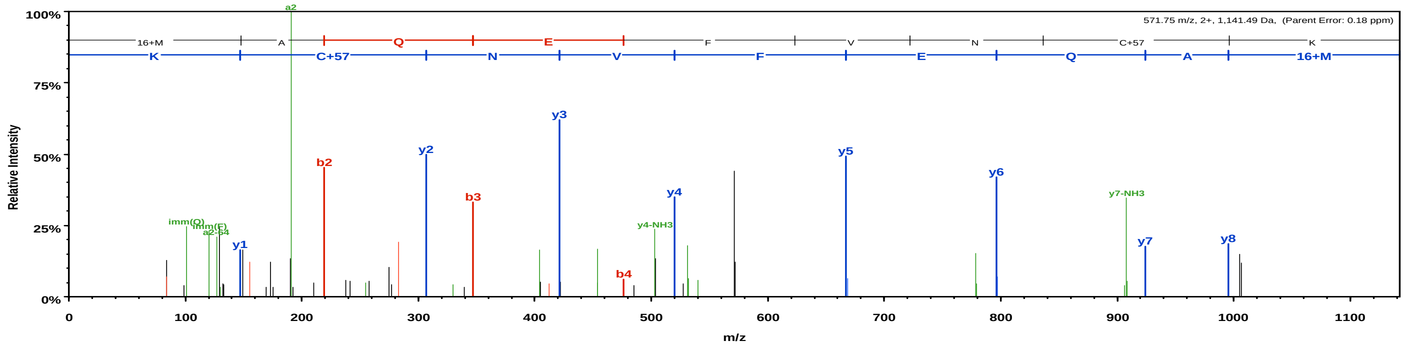

0 mM GSSG
Scan# 17240
XCorr: 2.53 (2+)

M(O)AQEFVNC(GSH)K

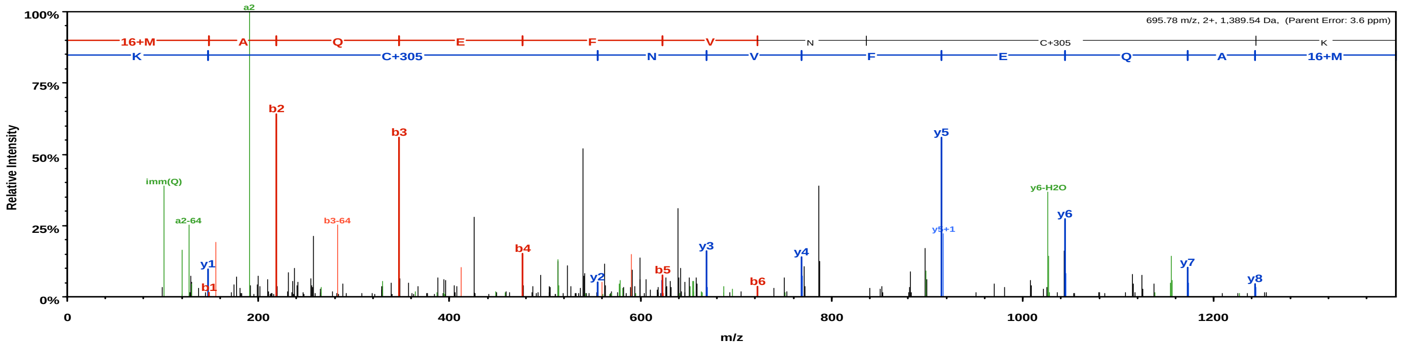

50 mM GSSG
Scan# 22534
XCorr: 2.75 (2+)

DC(CAM)IG(CAM)GCSDLISM(O)QQTGELM(O)TR

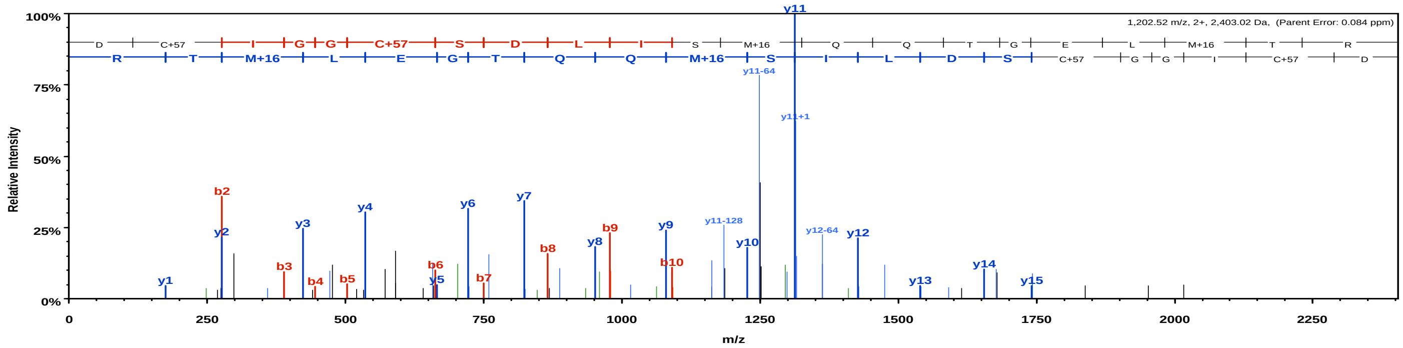

0 mM GSSG
QE+_0182
Scan# 37052
XCorr: 4.7 (2+)

DC(O3)IG(GSH)GCSDLISM(O)QQTGELM(O)TR

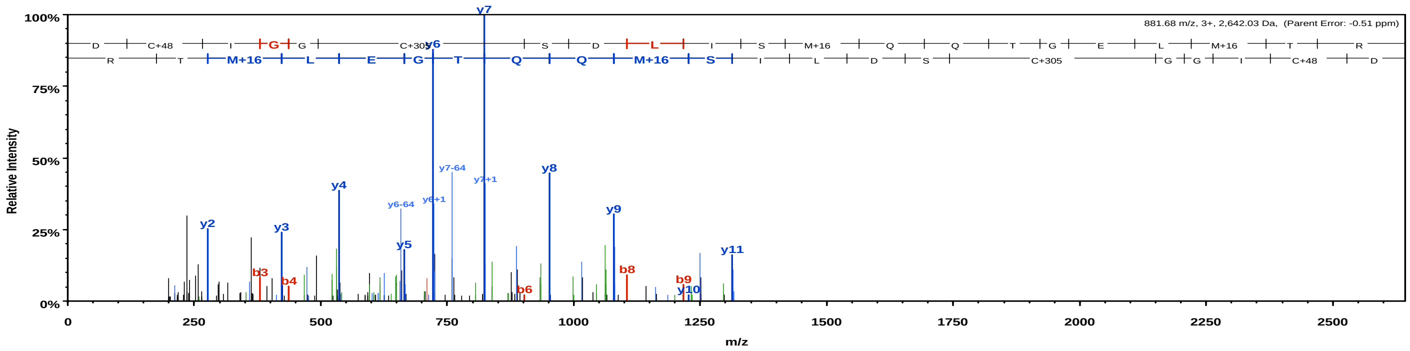

50 mM GSSG
QE+_0184
Scan# 40020
XCorr: 3.05 (3+)

Supplementary Figure 3

DC(GSH)IG(O3)GCSDLISM(O)QQTGELM(O)TR

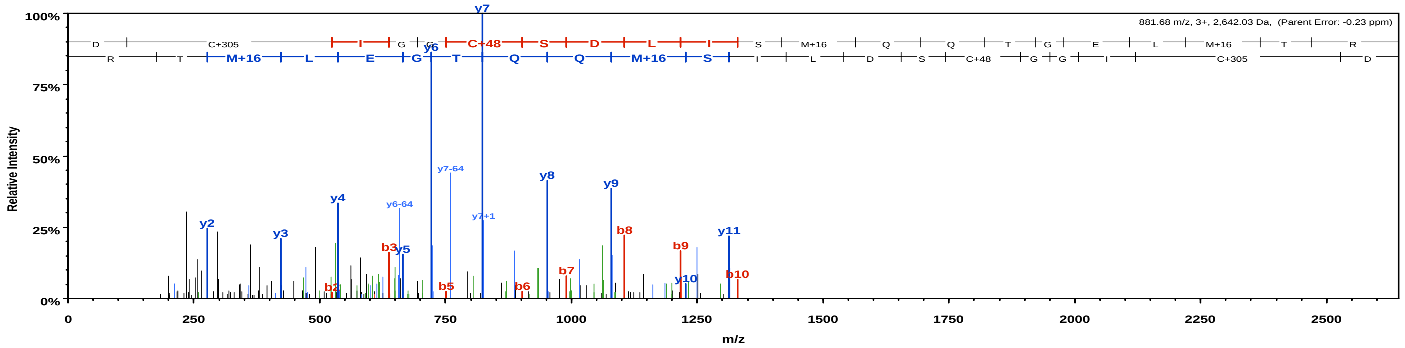

50 mM GSSG
QE+_0184
Scan# 38844
XCorr: 3.62 (3+)

DC(GSH)IG(GSH)GCSDLISM(O)QQTGELM(O)TR

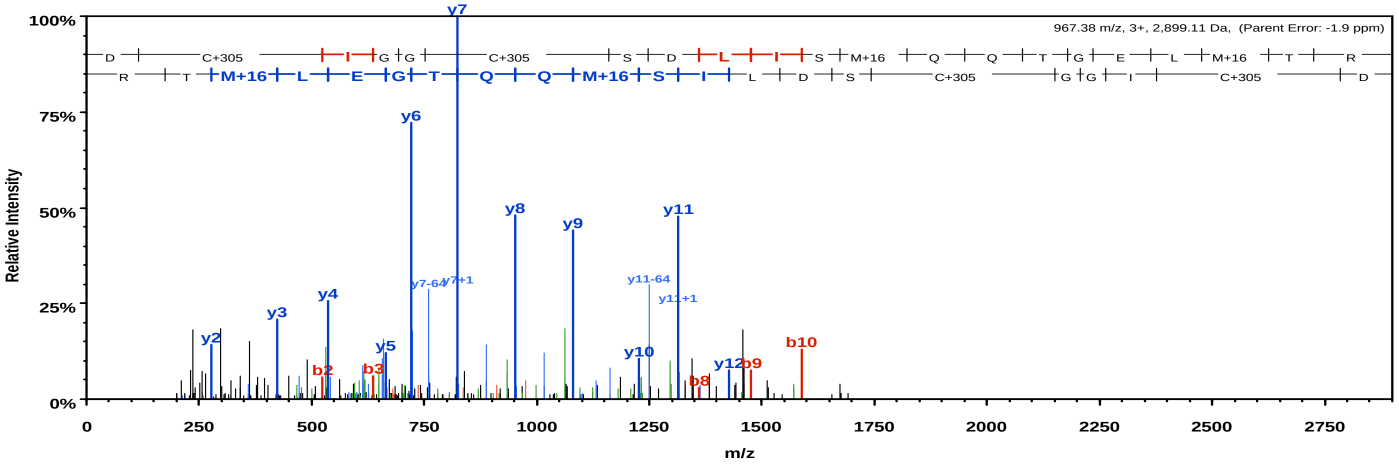

50 mM GSSG
QE+_0642
Scan# 16454
XCorr: 4.35 (3+)

Supplementary Figure 3

VVVFIKPTC(CAM)PYC(CAM)R

0 mM GSSG / QE+_0638 / Scan# 15846 / XCorr: 3.17 (3+)

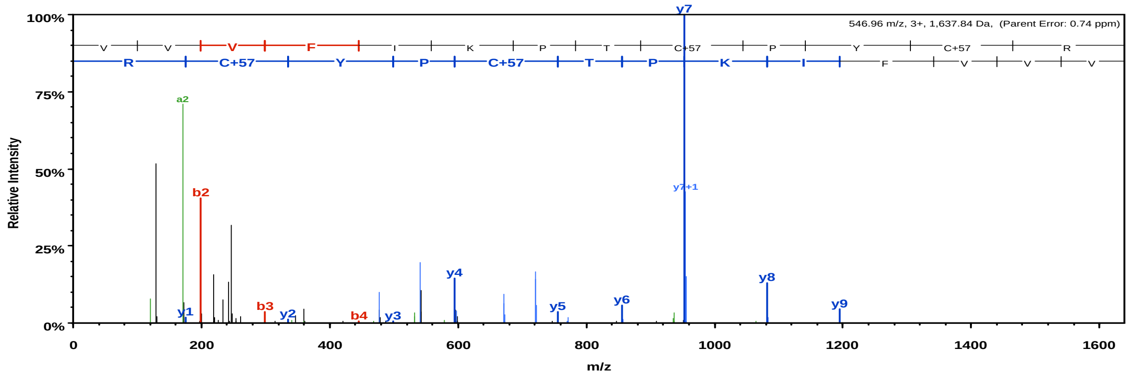

B	B Ions	B+2H	B-NH3	B-H2O	AA	Y Ions	Y+2H	Y-NH3	Y-H2O	Y
1	100.1	50.5			V	1,638.8	819.9	1,621.8	1,620.8	13
2	199.1	100.1			V	1,539.8	770.4	1,522.8	1,521.8	12
3	298.2	149.6			V	1,440.7	720.9	1,423.7	1,422.7	11
4	445.3	223.1			F	1,341.6	671.3	1,324.6	1,323.6	10
5	558.4	279.7			I	1,194.6	597.8	1,177.5	1,176.6	9
6	686.5	343.7	669.4		K	1,081.5	541.2	1,064.5	1,063.5	8
7	783.5	392.3	766.5		P	953.4	477.2	936.4	935.4	7
8	884.6	442.8	867.5	866.5	T	856.3	428.7	839.3	838.3	6
9	1,044.6	522.8	1,027.6	1,026.6	C+57	755.3	378.2	738.3		5
10	1,141.6	571.3	1,124.6	1,123.6	P	595.3	298.1	578.2		4
11	1,304.7	652.9	1,287.7	1,286.7	Y	498.2	249.6	481.2		3
12	1,464.7	732.9	1,447.7	1,446.7	C+57	335.1	168.1	318.1		2
13	1,638.8	819.9	1,621.8	1,620.8	R	175.1	88.1	158.1		1

VVVFIKPTC(GSH)PYC(O3)R

1 mM GSSG / QE+_0640 / Scan# 16548 / XCorr: 2.97 (2+)

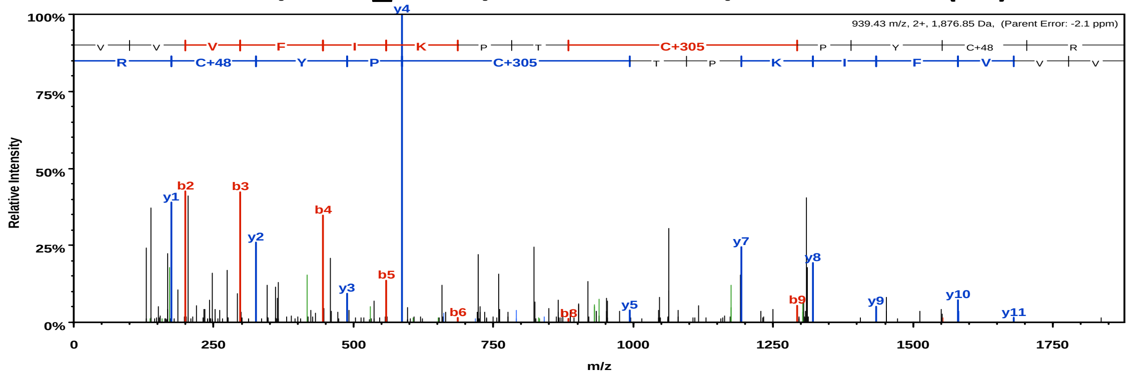

B	B Ions	B+2H	B-NH3	B-H2O	AA	Y Ions	Y+2H	Y-NH3	Y-H2O	Y
1	100.1				V	1,877.9	939.4	1,860.8	1,859.8	13
2	199.1				V	1,778.8	889.9	1,761.8	1,760.8	12
3	298.2				V	1,679.7	840.4	1,662.7	1,661.7	11
4	445.3				F	1,580.7	790.8	1,563.6	1,562.6	10
5	558.4				I	1,433.6	717.3	1,416.6	1,415.6	9
6	686.5	343.7	669.4		K	1,320.5	660.8	1,303.5	1,302.5	8
7	783.5	392.3	766.5		P	1,192.4	596.7	1,175.4	1,174.4	7
8	884.6	442.8	867.5	866.5	T	1,095.4	548.2	1,078.3	1,077.3	6
9	1,292.6	646.8	1,275.6	1,274.6	C+305	994.3		977.3		5
10	1,389.7	695.3	1,372.7	1,371.7	P	586.2		569.2		4
11	1,552.8	776.9	1,535.7	1,534.7	Y	489.2		472.1		3
12	1,703.7	852.4	1,686.7	1,685.7	C+48	326.1		309.1		2
13	1,877.9	939.4	1,860.8	1,859.8	R	175.1		158.1		1

VVVFIKPTC(O3)PYC(GSH)R

50 mM GSSG / QE+_0642 / Scan# 16668/ XCorr: 3.26 (2+)

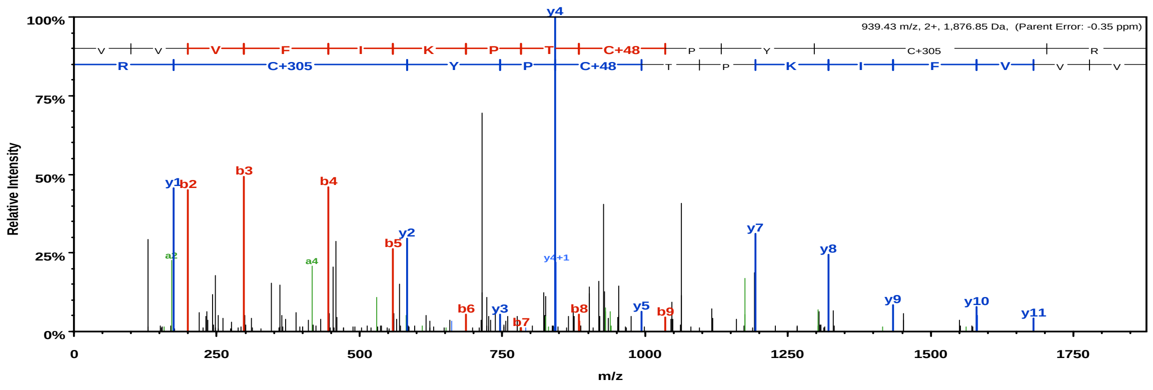

B	B Ions	B+2H	B-NH3	B-H2O	AA	Y Ions	Y+2H	Y-NH3	Y-H2O	Y
1	100.1				V	1,877.9	939.4	1,860.8	1,859.8	13
2	199.1				V	1,778.8	889.9	1,761.8	1,760.8	12
3	298.2				V	1,679.7	840.4	1,662.7	1,661.7	11
4	445.3				F	1,580.7	790.8	1,563.6	1,562.6	10
5	558.4				I	1,433.6	717.3	1,416.6	1,415.6	9
6	686.5	343.7	669.4		K	1,320.5	660.8	1,303.5	1,302.5	8
7	783.5	392.3	766.5		P	1,192.4	596.7	1,175.4	1,174.4	7
8	884.6	442.8	867.5	866.5	T	1,095.4	548.2	1,078.3	1,077.3	6
9	1,035.6	518.3	1,018.5	1,017.5	C+48	994.3		977.3		5
10	1,132.6	566.8	1,115.6	1,114.6	P	843.3		826.3		4
11	1,295.7	648.3	1,278.6	1,277.7	Y	746.3		729.2		3
12	1,703.7	852.4	1,686.7	1,685.7	C+305	583.2		566.2		2
13	1,877.9	939.4	1,860.8	1,859.8	R	175.1		158.1		1

Supplementary Figure 4

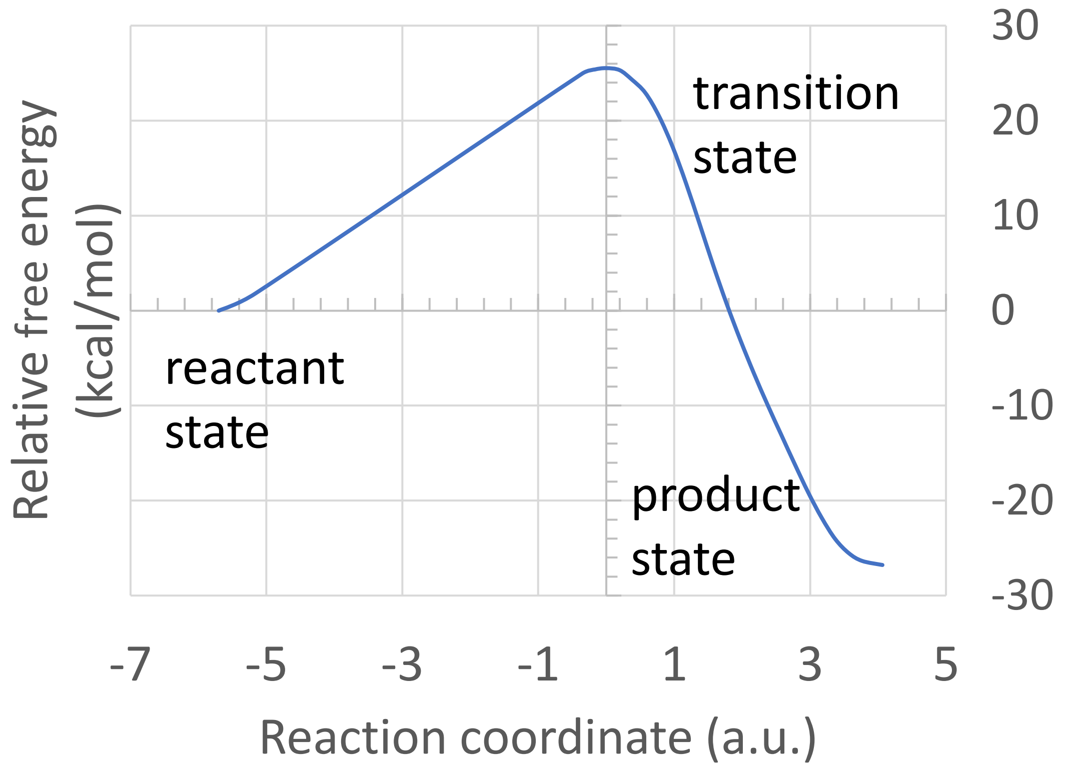

Supplementary Figure 5

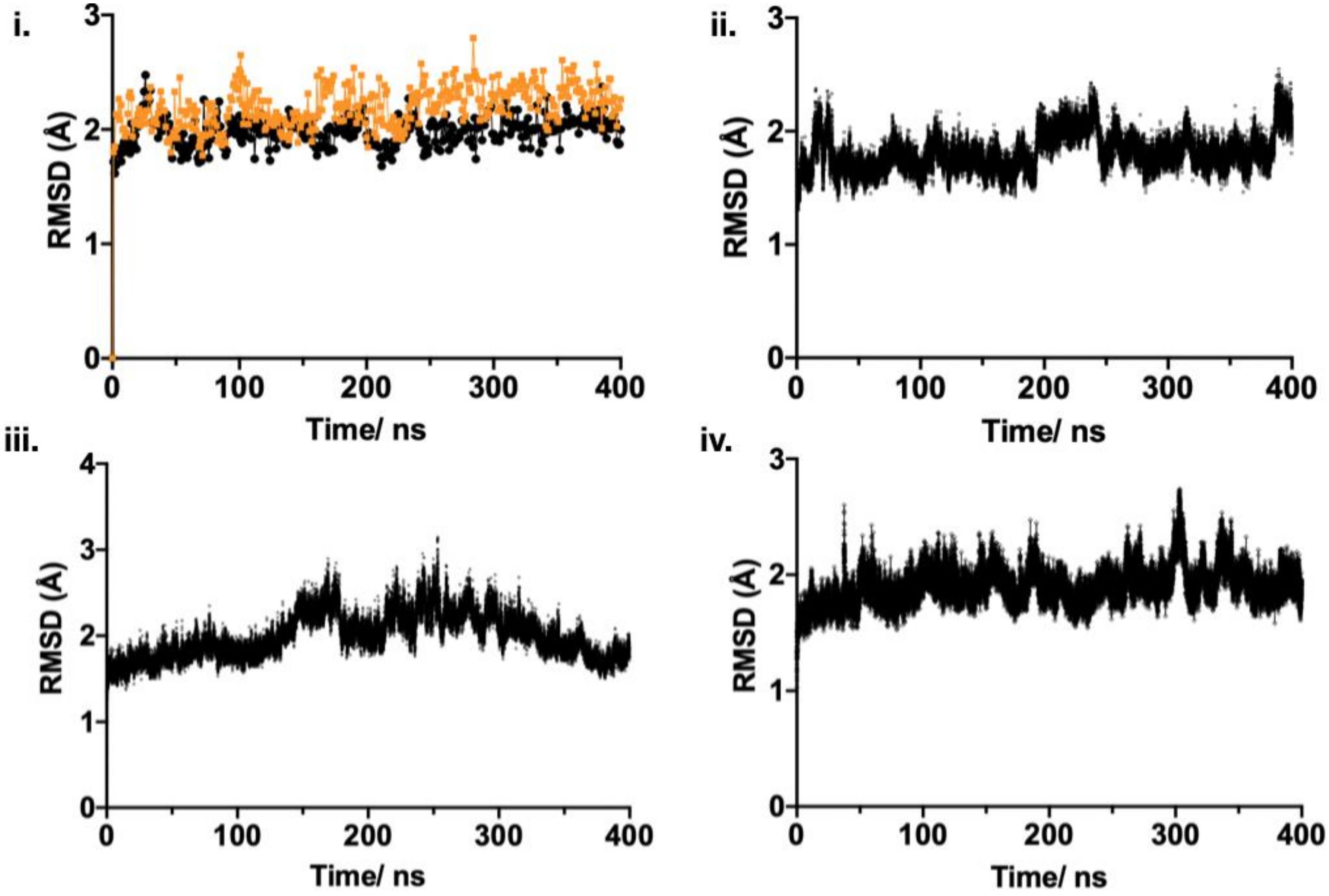

Supplementary Figure 6

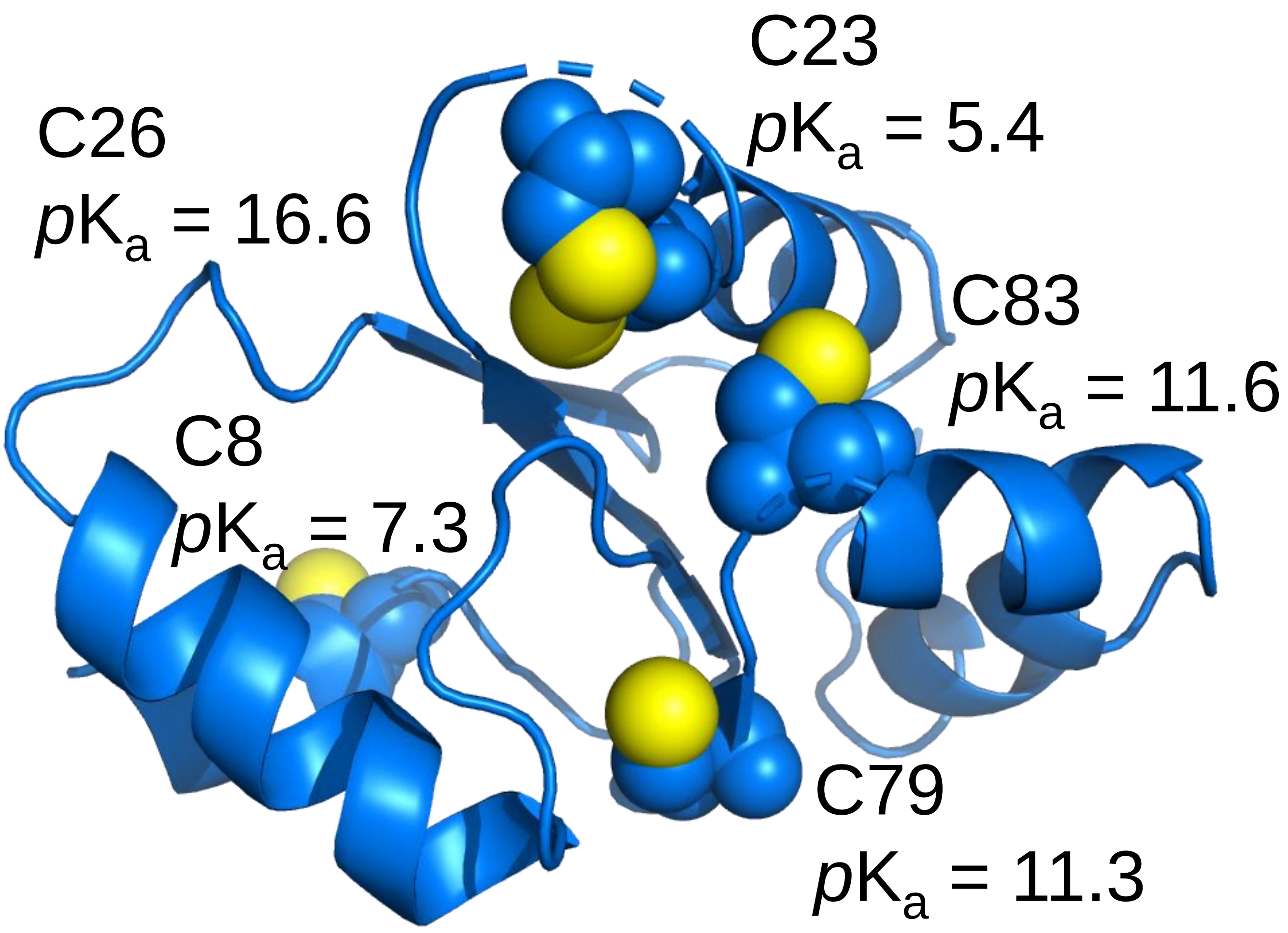

Supplementary Figure 7

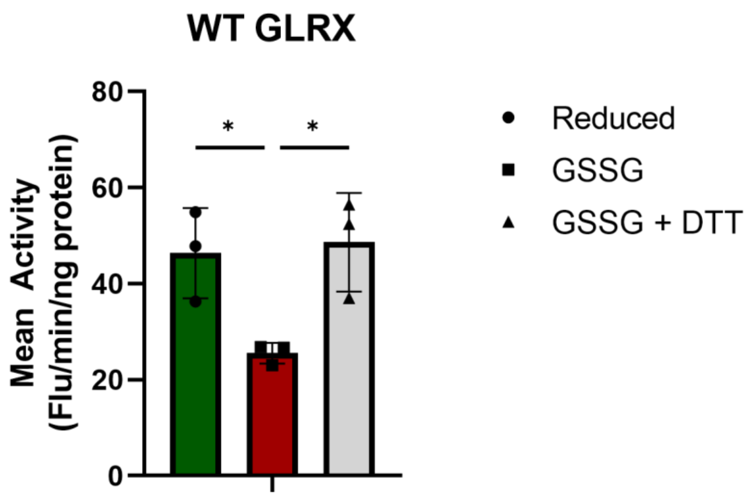

Supplementary Figure 8

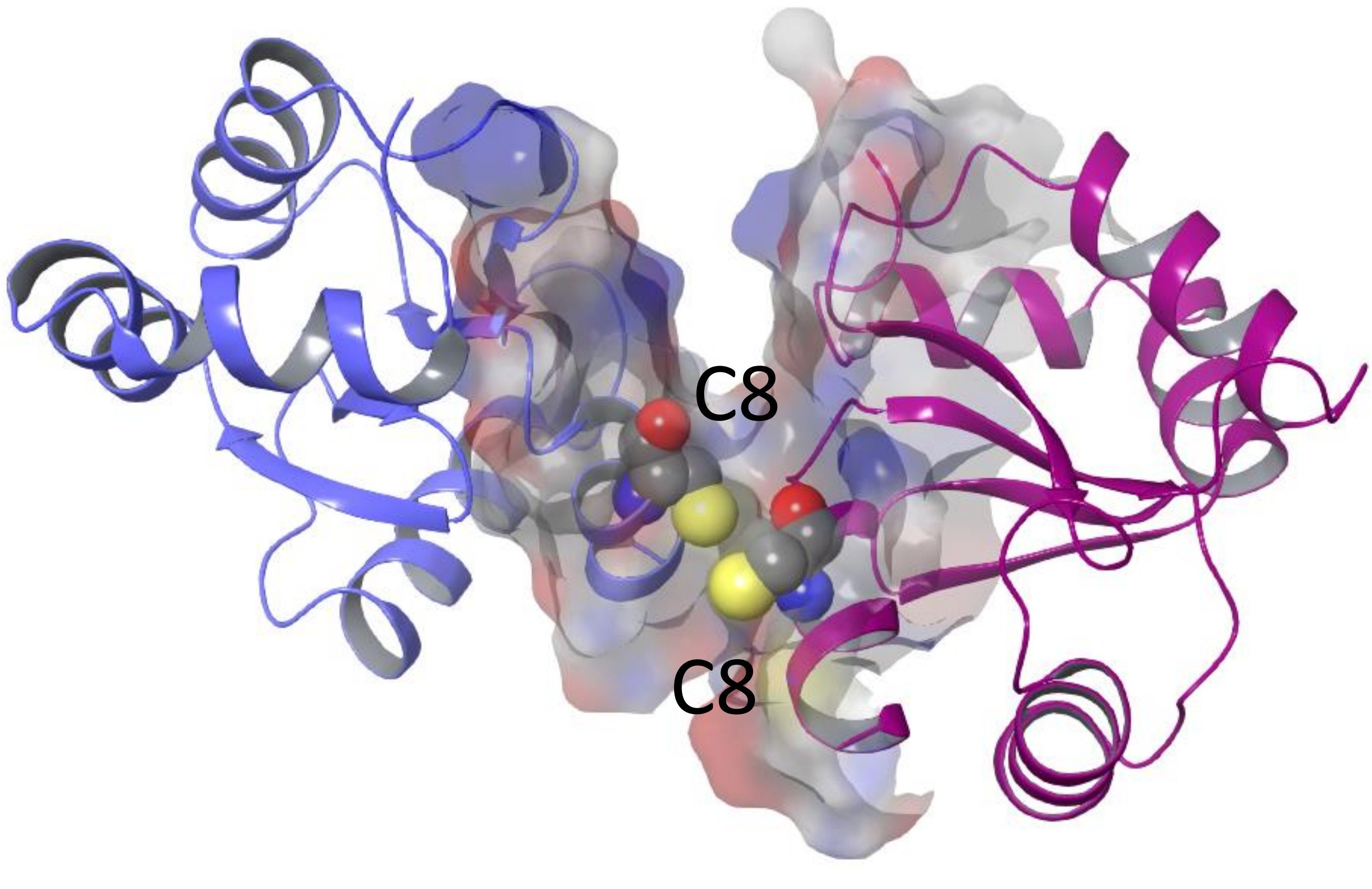

Supplementary Figure 9

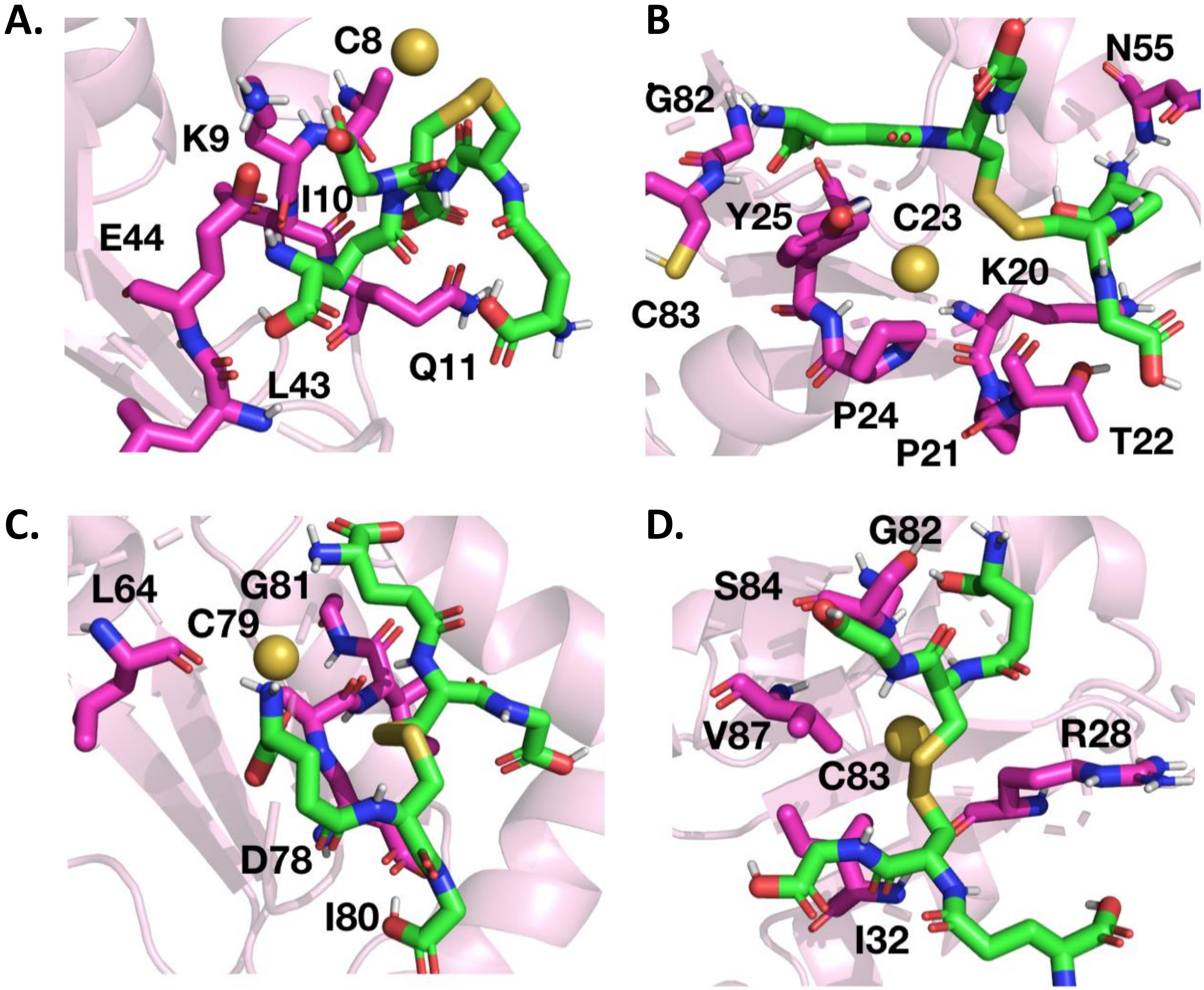

Supplementary Figure 10

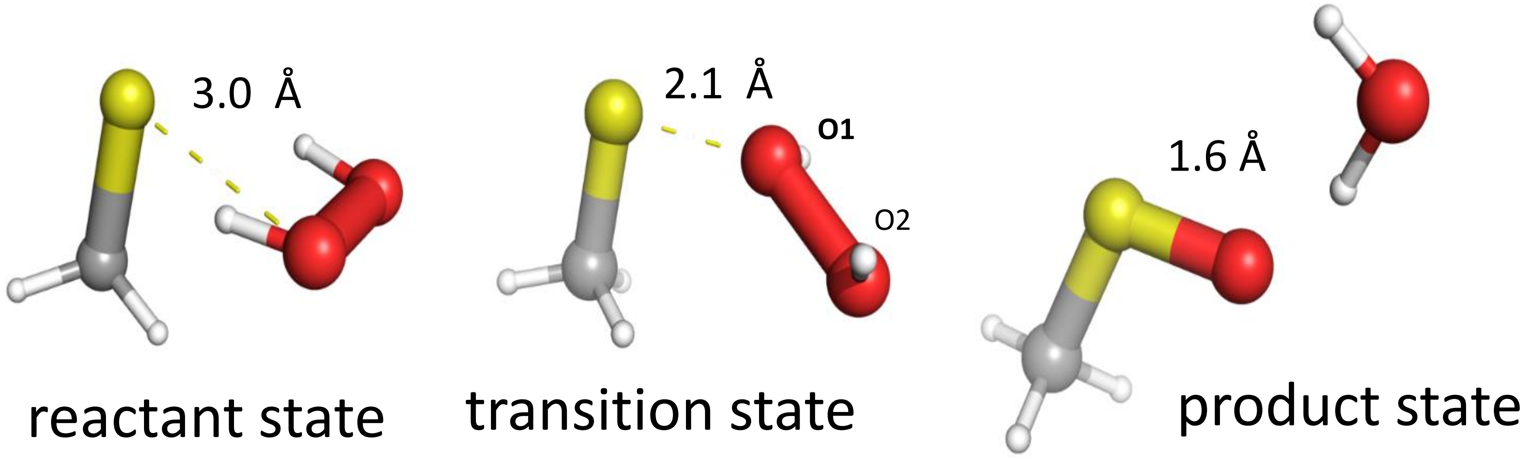

v = -262.68 cm^{-1}

Supplementary Figure 11

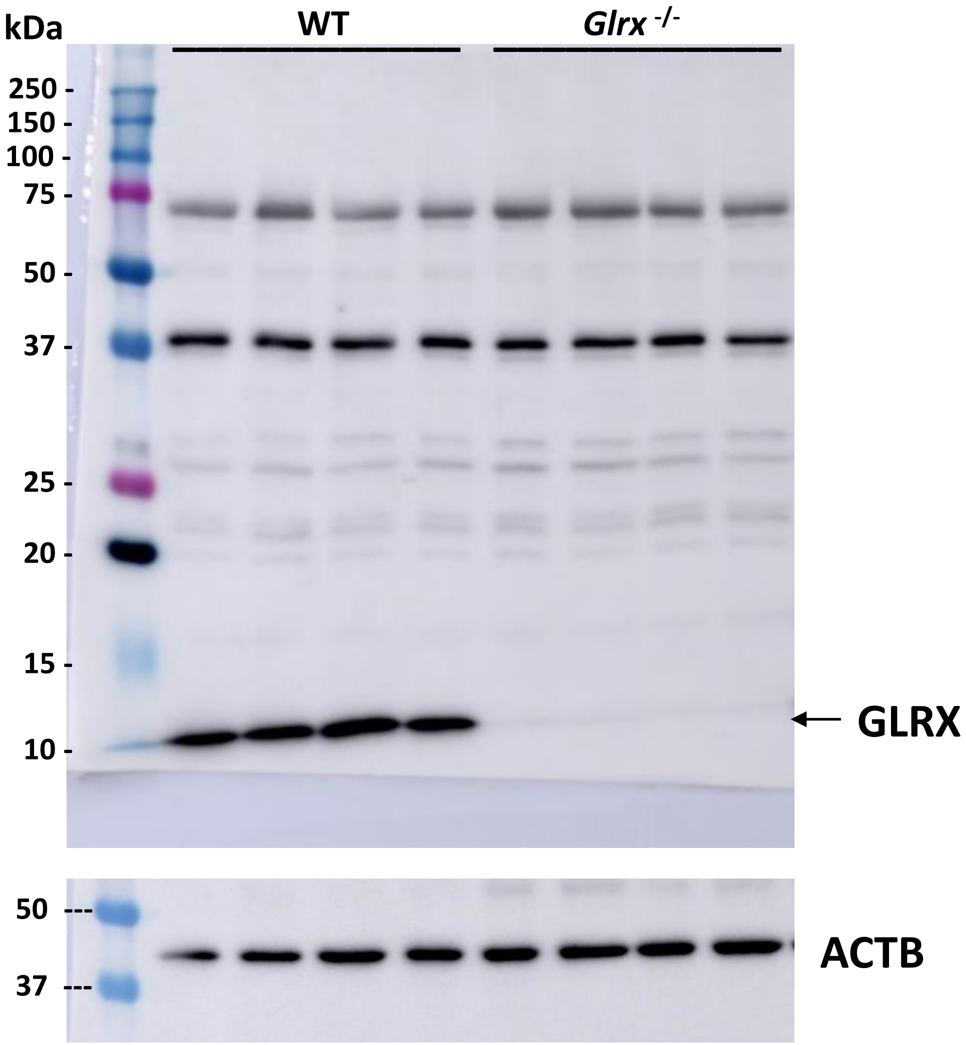

Supplement: Supplementary file 1 — Supplementary Information [file 41467_2023_39664_MOESM1_ESM.pdf]
