# Supplementary material for: Structural and functional fine mapping of cysteines in mammalian glutaredoxin reveal their differential oxidation susceptibility
Source: Nat Commun. 2023 Jul 28;14:4550. doi: 10.1038/s41467-023-39664-2 (PMC10382592; doi:10.1038/s41467-023-39664-2)
Supplement: Supplementary file 3 — Description of Additional Supplementary Material Files [file 41467_2023_39664_MOESM3_ESM.pdf]

**Description of Additional Supplementary Material**

**File name:** Supplementary Data 1

**Description:** Uncropped gels and blots

**File name:** Supplementary Movie 1

**Description:** 200-ns MD simulation of GLRX monomer (PDB ID: 4RQR) aligned to a zebrafish GLRX2 dimeric structure in solution. Each monomer is shown in different color and C8 involved in symmetric interface is labelled.

**File name:** Supplementary Movie 2

**Description:** 200-ns MD simulation of three free GLRX monomers in solution. Each monomer is shown in different color and the cysteines involved in symmetric and asymmetric interfaces are labelled.
