# Supplementary material for: Structural and functional fine mapping of cysteines in mammalian glutaredoxin reveal their differential oxidation susceptibility
Source: Nat Commun. 2023 Jul 28;14:4550. doi: 10.1038/s41467-023-39664-2 (PMC10382592; doi:10.1038/s41467-023-39664-2)

Figure 6 – Replicate 1, blot in manuscript, uncropped

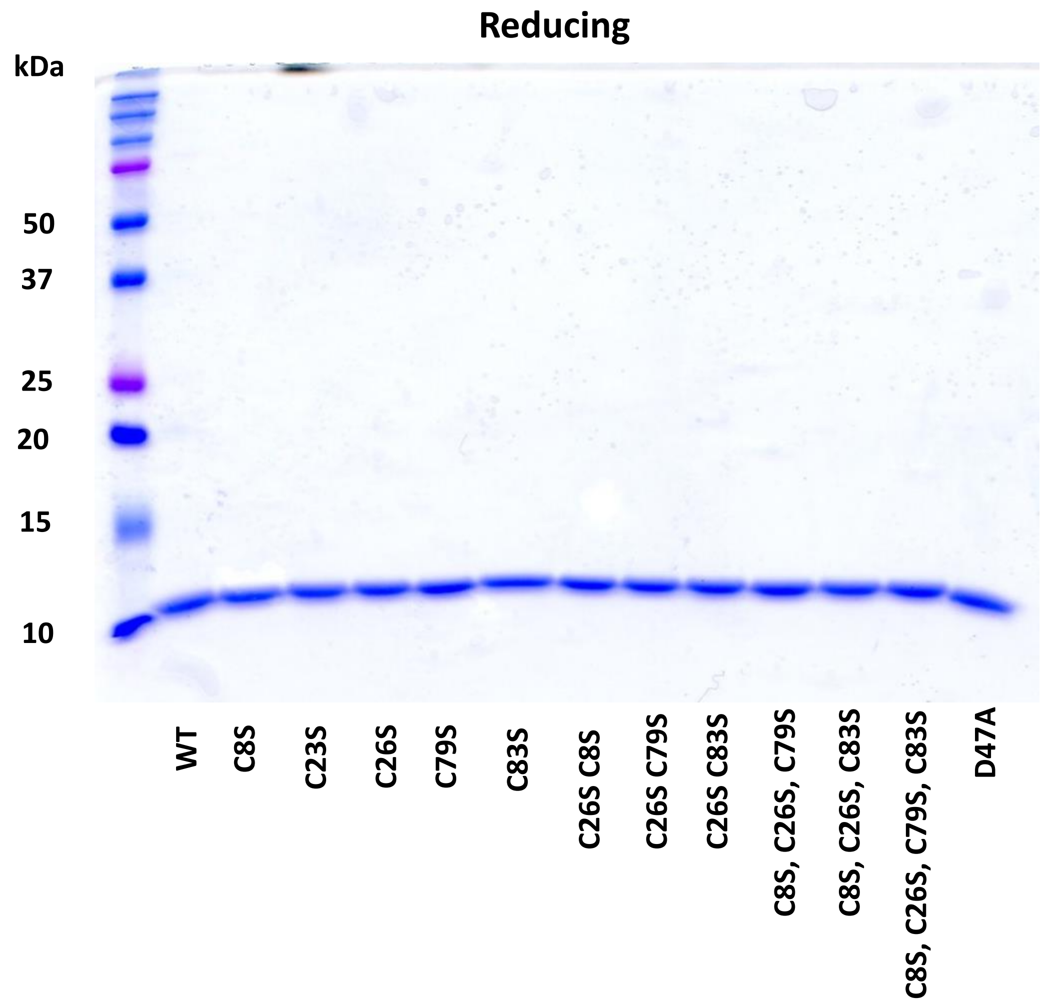

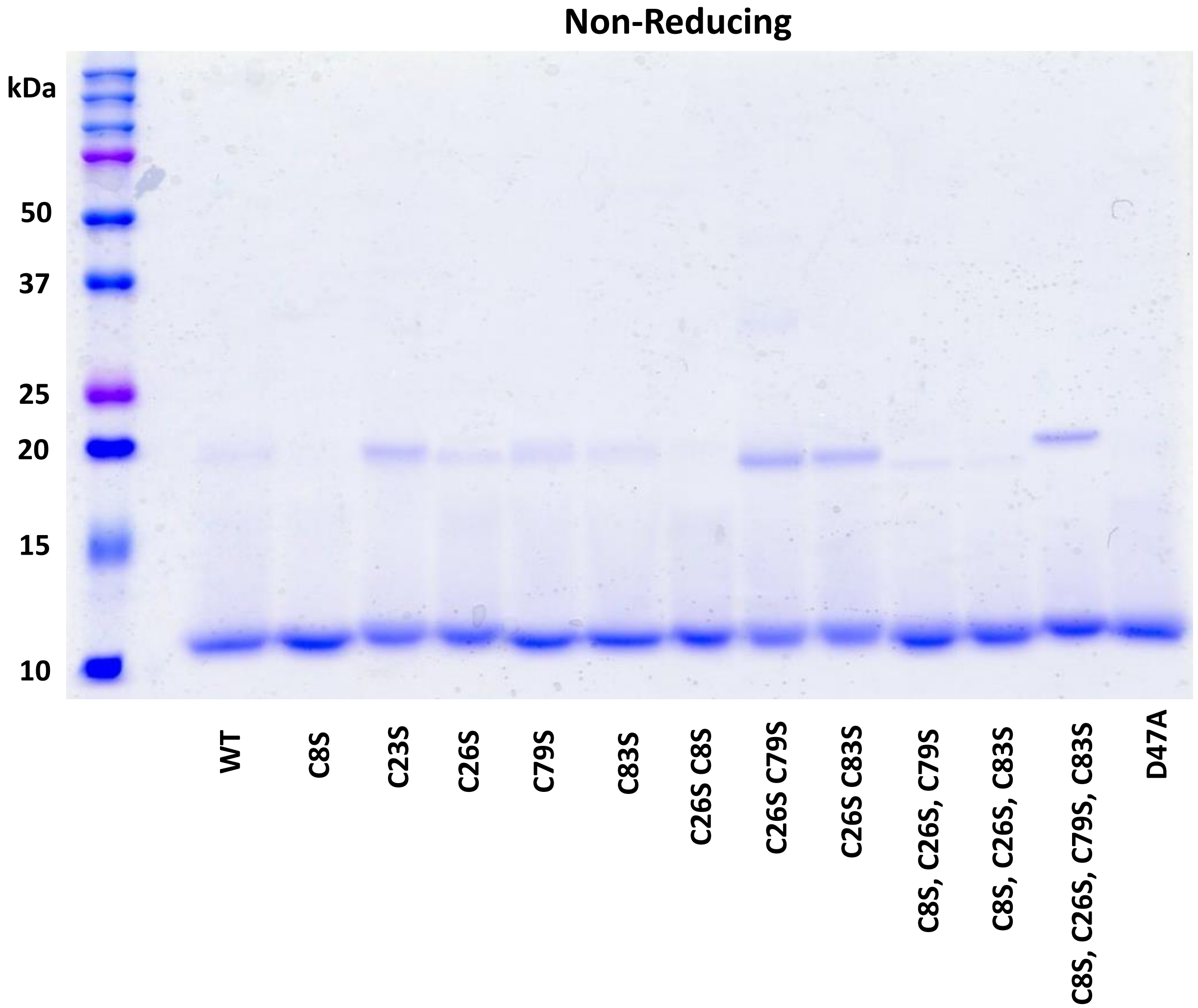

Figure 6 – Replicate 2A

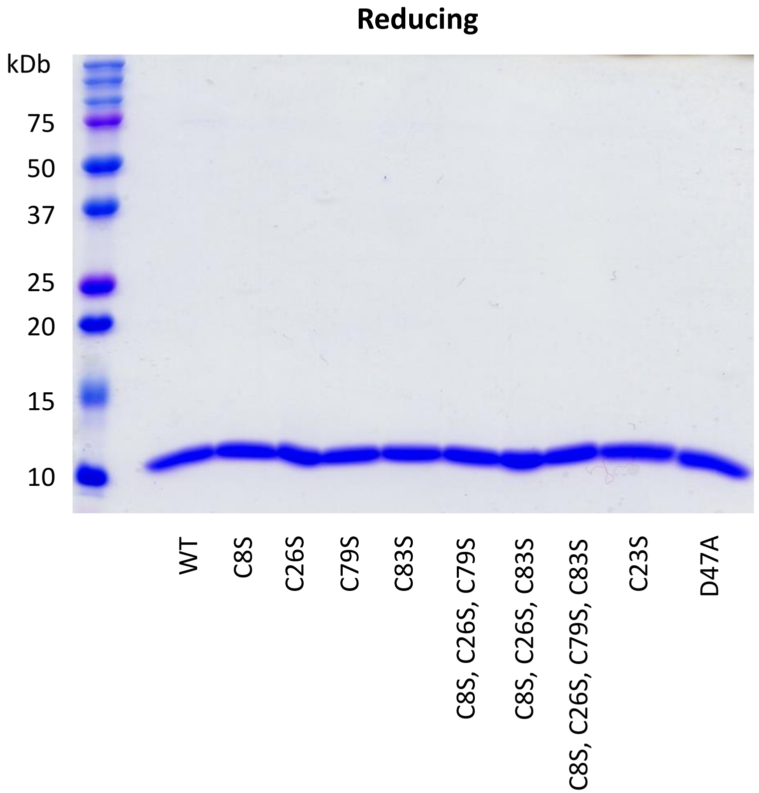

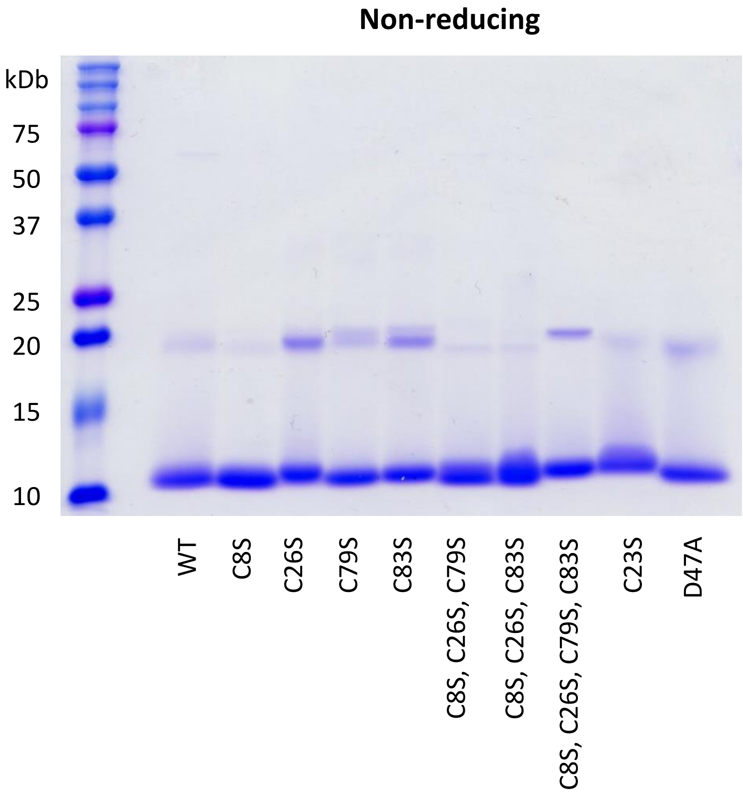

Figure 6 – Replicate 2B

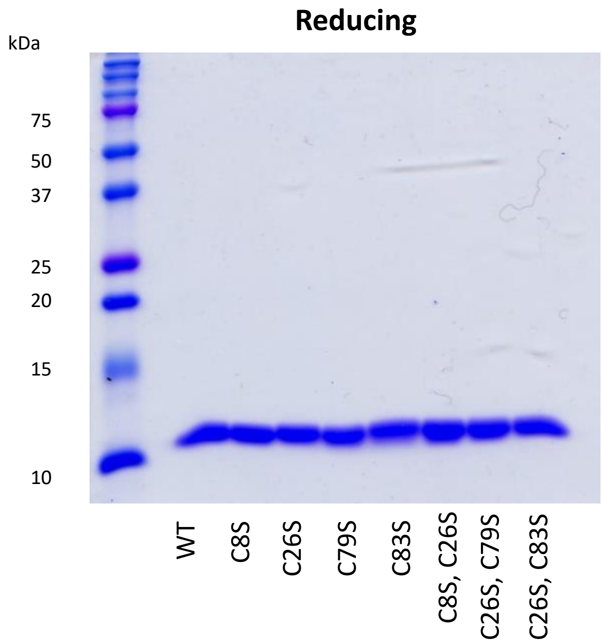

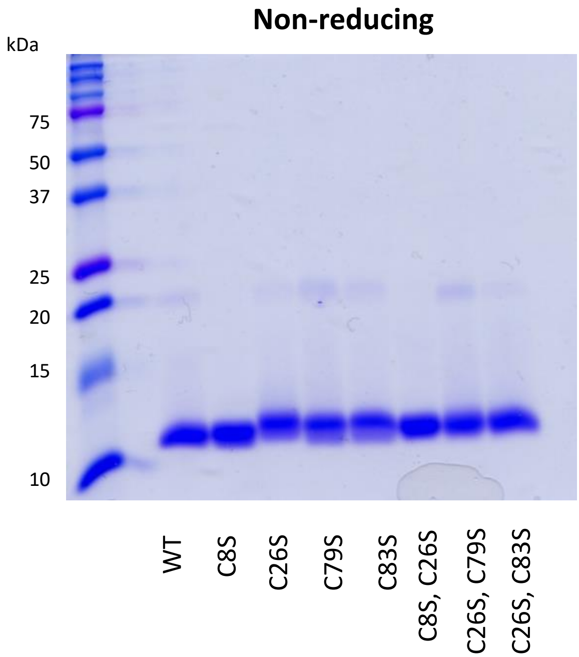

Figure 6 – Replicate 3A

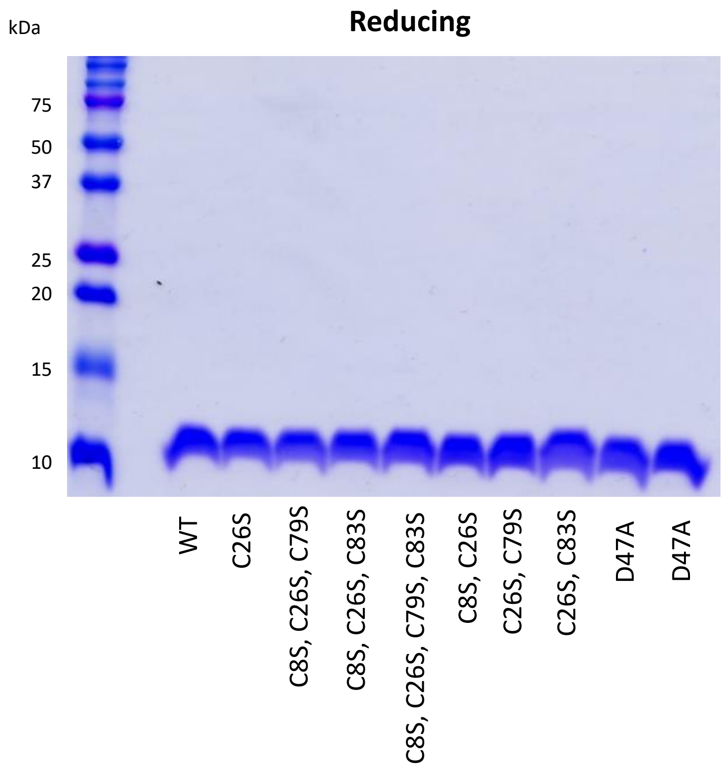

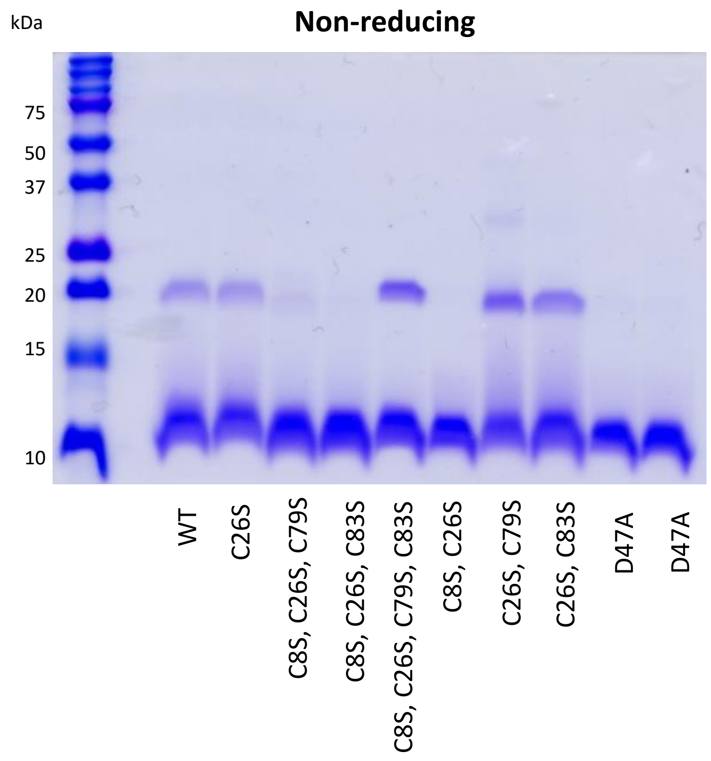

Figure 6 – Replicate 3B

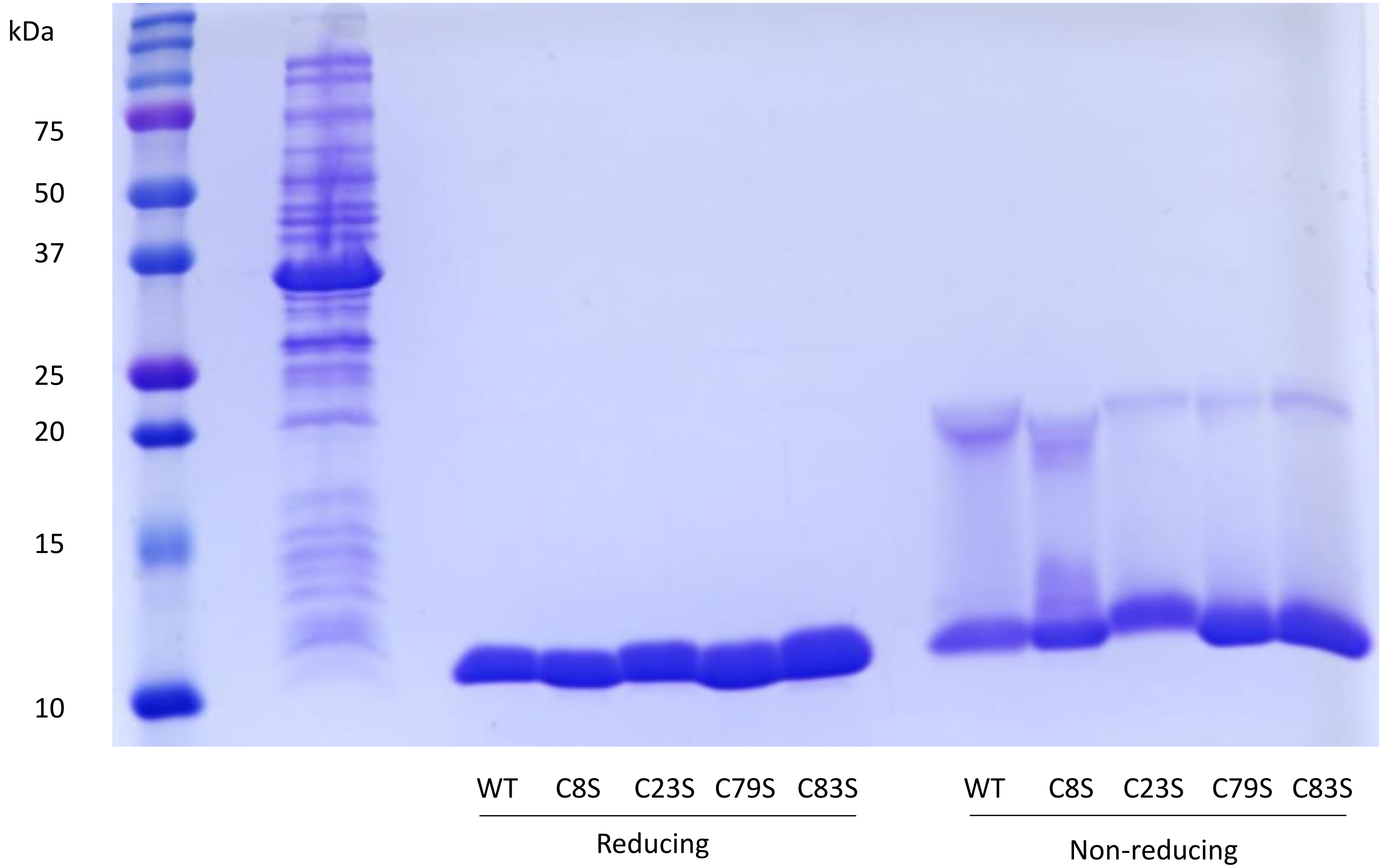

Figure 7A – Replicate 1, shown in manuscript

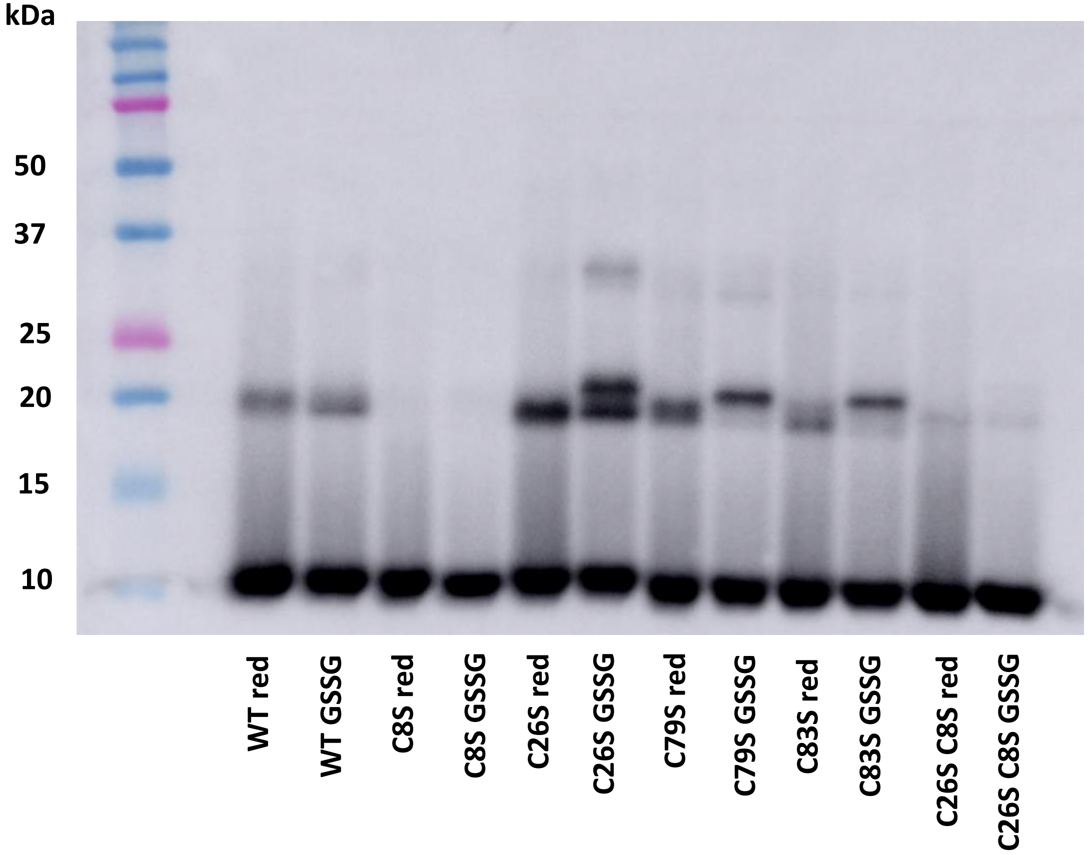

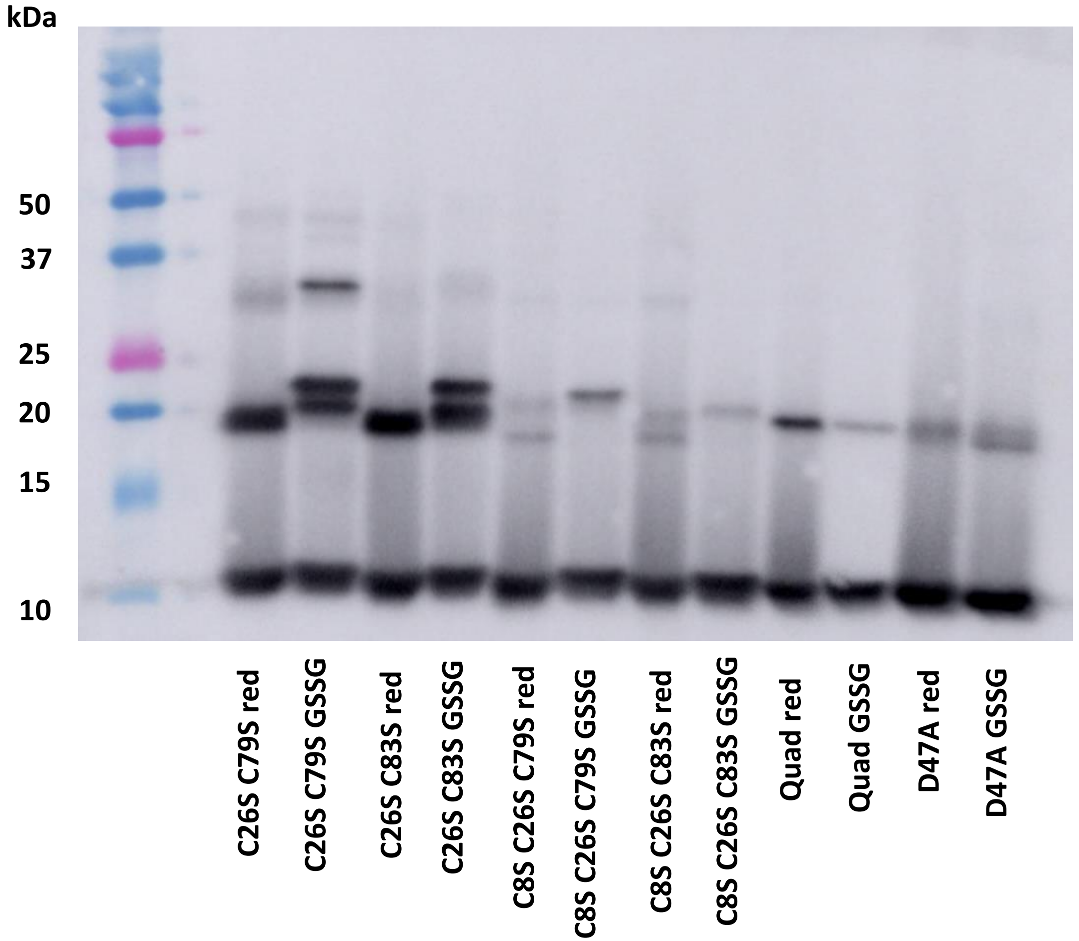

Figure 7A – replicate 2

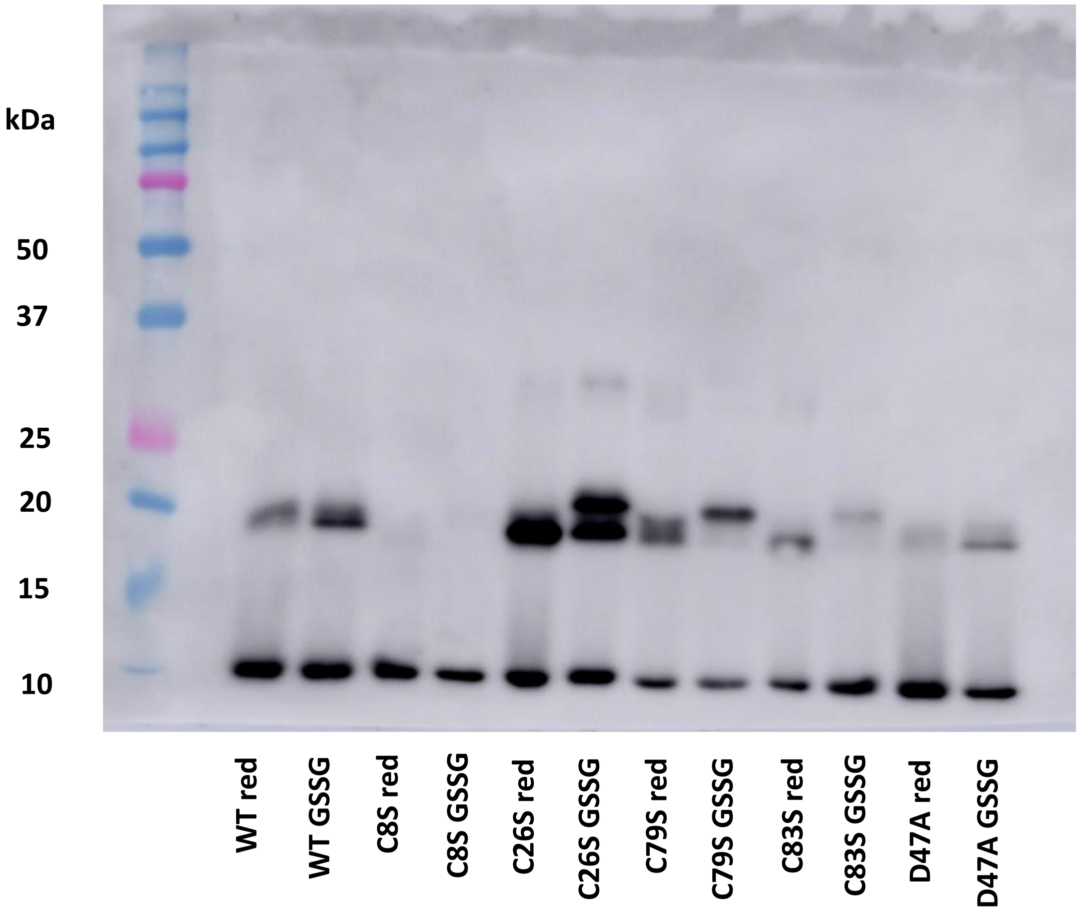

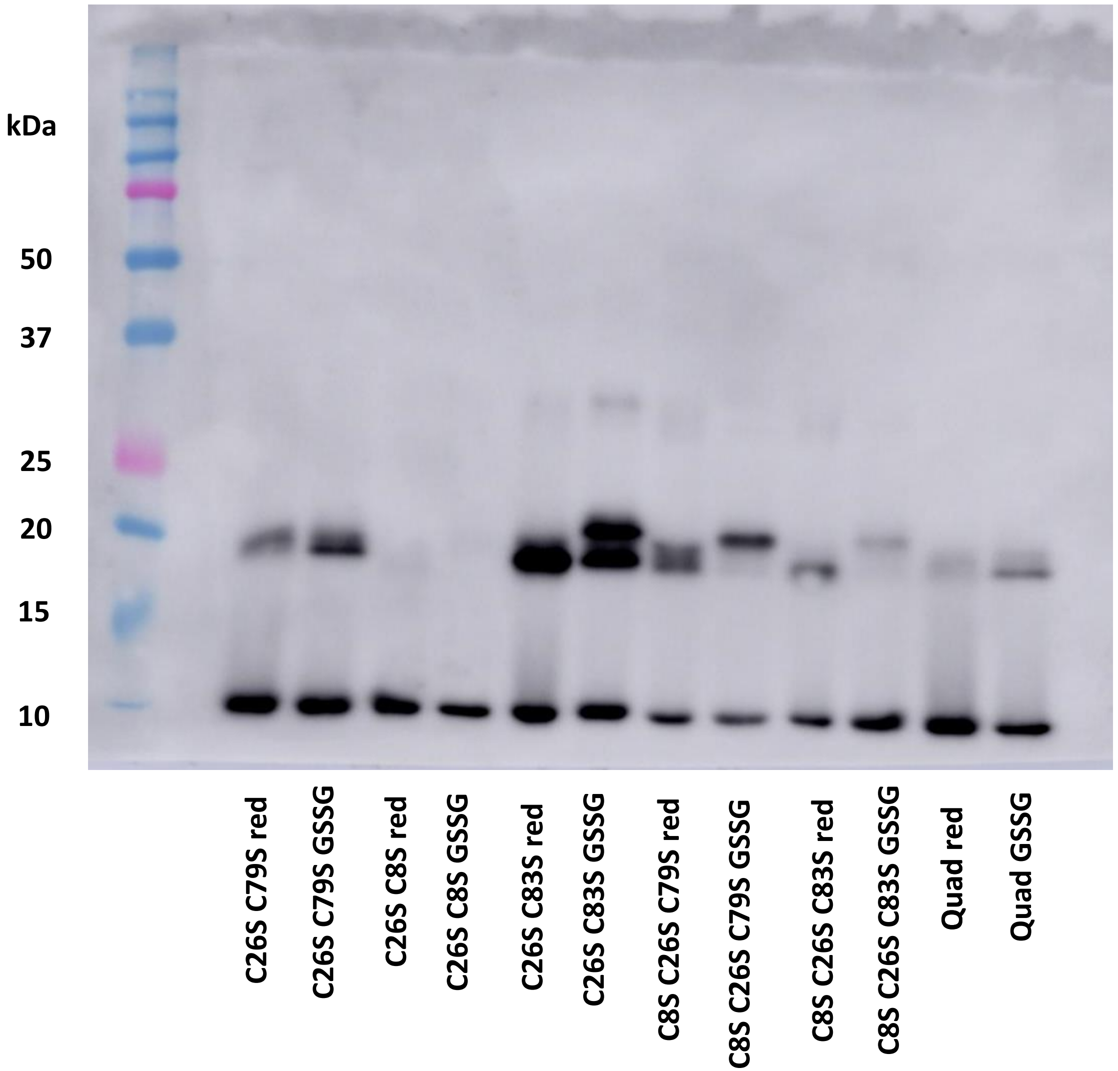

Figure 7A – replicate 3

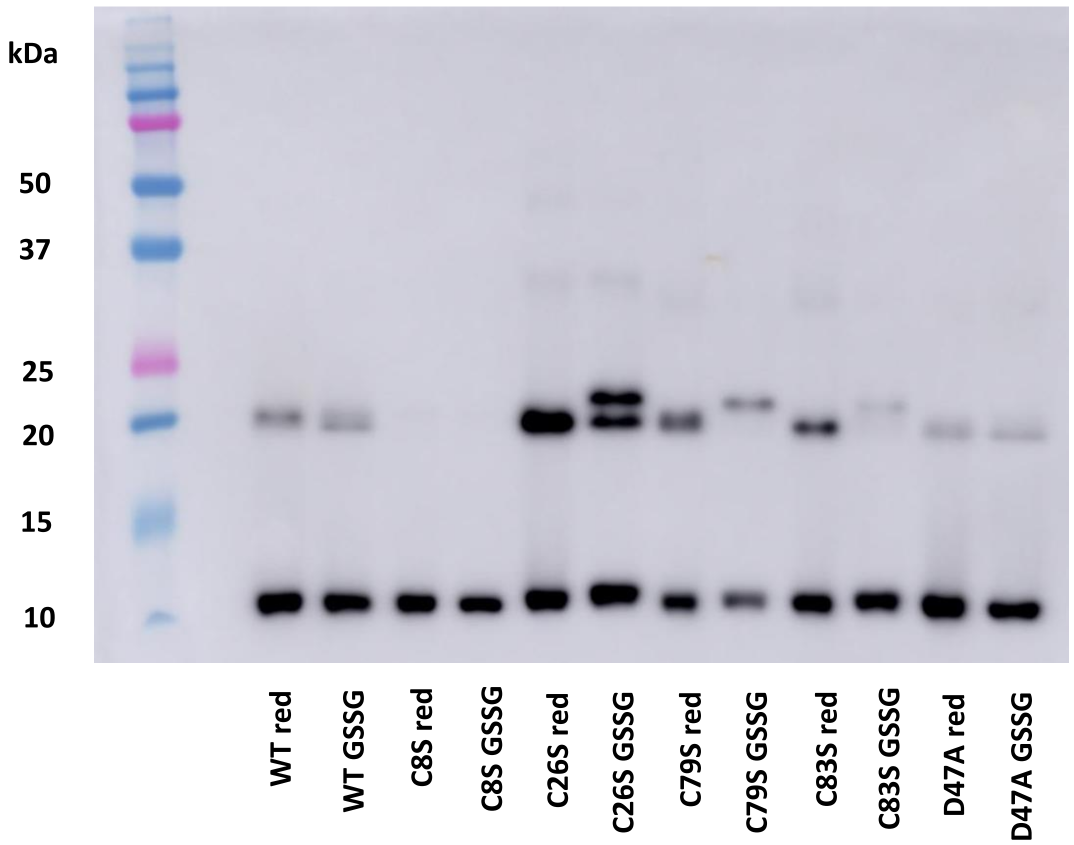

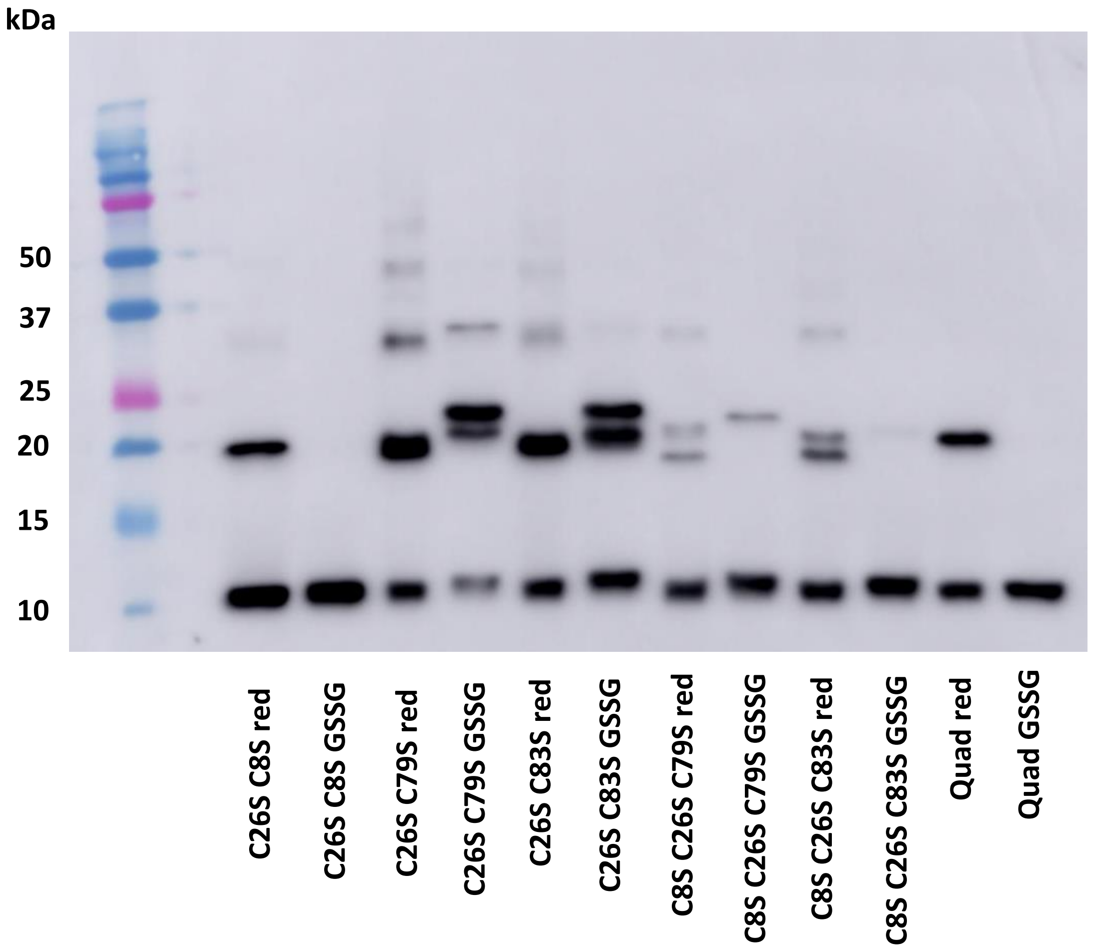

Figure 7B – Replicate 1, shown in manuscript

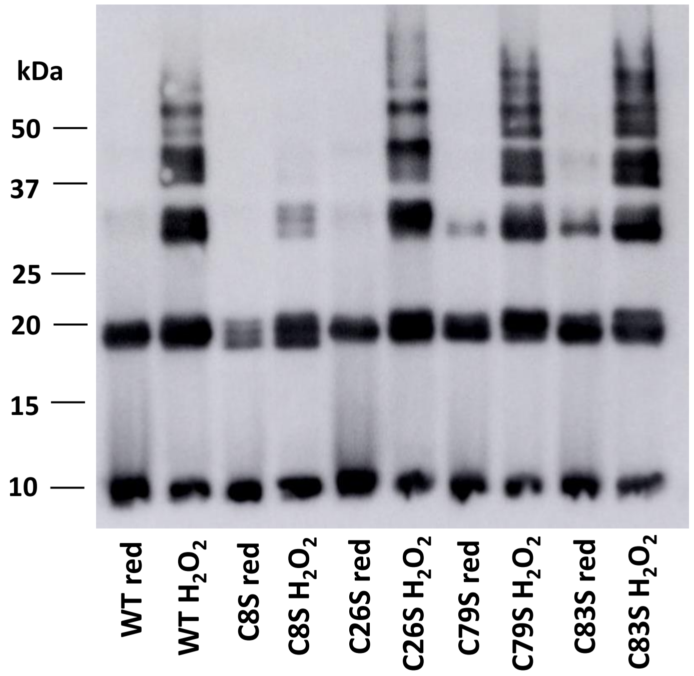

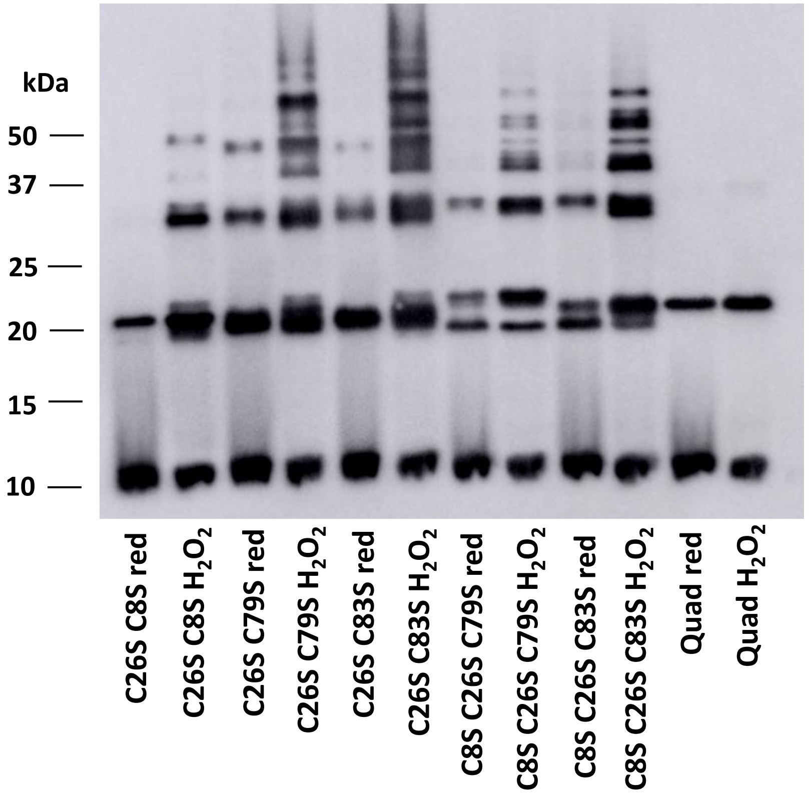

Figure 7B – Replicate 2

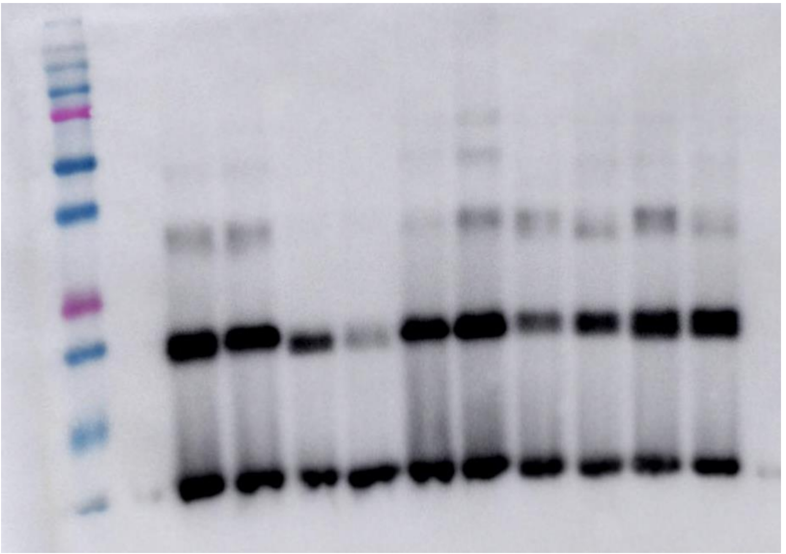

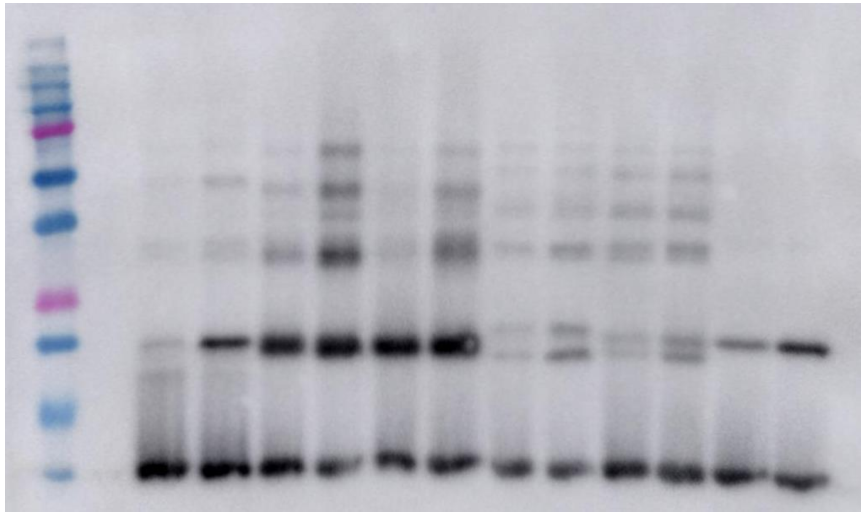

Figure 7B – Replicate 3

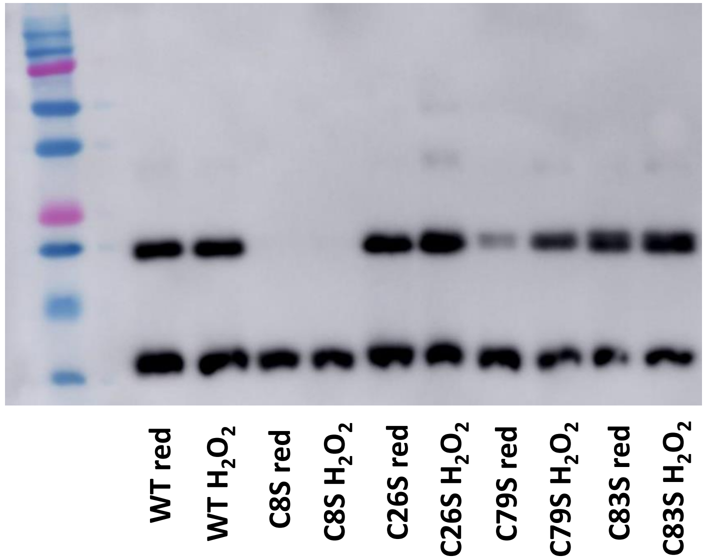

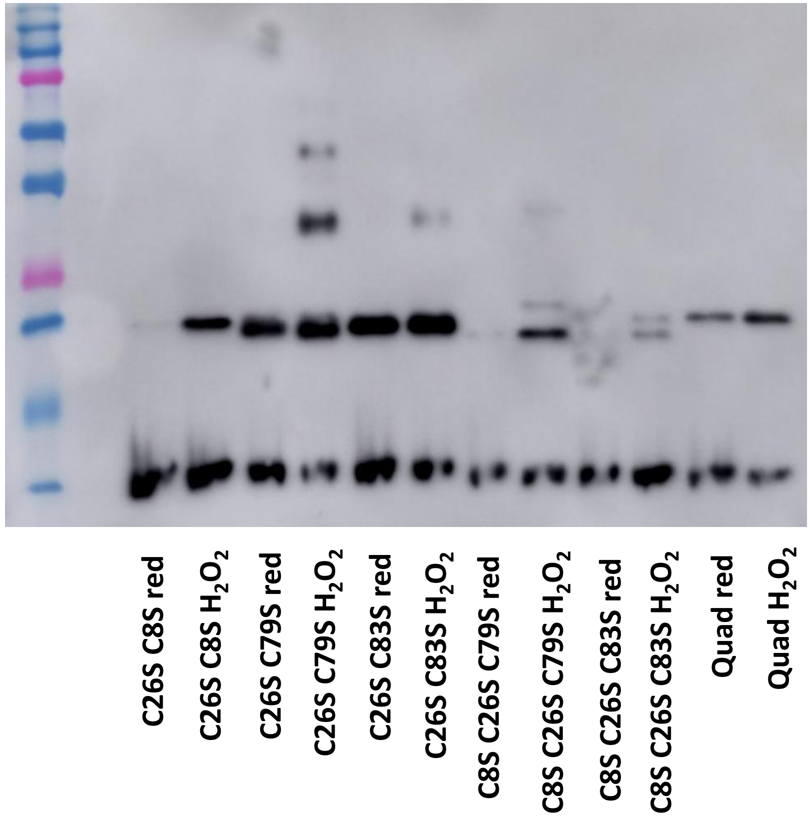

Supplement: Supplementary file 4 — Supplementary Data 1 [file 41467_2023_39664_MOESM4_ESM.pdf]
