# Supplementary material for: Structural and functional fine mapping of cysteines in mammalian glutaredoxin reveal their differential oxidation susceptibility
Source: Nat Commun. 2023 Jul 28;14:4550. doi: 10.1038/s41467-023-39664-2 (PMC10382592; doi:10.1038/s41467-023-39664-2)
Supplement: Supplementary file 7 — Reporting Summary [file 41467_2023_39664_MOESM7_ESM.pdf]

nature portfolio

Corresponding author(s): Yvonne Janssen-Heininger, Jianing Li

Last updated by author(s): Jun 1, 2023

Reporting Summary

Nature Portfolio wishes to improve the reproducibility of the work that we publish. This form provides structure for consistency and transparency in reporting. For further information on Nature Portfolio policies, see our Editorial Policies and the Editorial Policy Checklist.

Statistics

For all statistical analyses, confirm that the following items are present in the figure legend, table legend, main text, or Methods section.

n/a	Confirmed	
☐	☒	The exact sample size (*n*) for each experimental group/condition, given as a discrete number and unit of measurement
☐	☒	A statement on whether measurements were taken from distinct samples or whether the same sample was measured repeatedly
☐	☒	The statistical test(s) used AND whether they are one- or two-sided<br>*Only common tests should be described solely by name; describe more complex techniques in the Methods section.*
☒	☐	A description of all covariates tested
☐	☒	A description of any assumptions or corrections, such as tests of normality and adjustment for multiple comparisons
☐	☒	A full description of the statistical parameters including central tendency (e.g. means) or other basic estimates (e.g. regression coefficient) AND variation (e.g. standard deviation) or associated estimates of uncertainty (e.g. confidence intervals)
☐	☒	For null hypothesis testing, the test statistic (e.g. *F*, *t*, *r*) with confidence intervals, effect sizes, degrees of freedom and *P* value noted<br>*Give P values as exact values whenever suitable.*
☒	☐	For Bayesian analysis, information on the choice of priors and Markov chain Monte Carlo settings
☒	☐	For hierarchical and complex designs, identification of the appropriate level for tests and full reporting of outcomes
☒	☐	Estimates of effect sizes (e.g. Cohen's *d*, Pearson's *r*), indicating how they were calculated

Our web collection on statistics for biologists contains articles on many of the points above.

Software and code

Policy information about availability of computer code

Data collection	All the computer simulations and calculations were carried out with software programs in the Schrödinger suite. MD simulations was run with Desmond, Induced-Fit Docking with IFD, Quantum chemistry calculations of the model system with Jaguar, and QM/MM calculations with Qsite.
Data analysis	Visualization was done with Maestro and Pymol (version 2.5, Schrödinger, Inc.) and data analysis was done with in-house Python scripts.

For manuscripts utilizing custom algorithms or software that are central to the research but not yet described in published literature, software must be made available to editors and reviewers. We strongly encourage code deposition in a community repository (e.g. GitHub). See the Nature Portfolio guidelines for submitting code & software for further information.

Data

Policy information about availability of data

All manuscripts must include a data availability statement. This statement should provide the following information, where applicable:

- Accession codes, unique identifiers, or web links for publicly available datasets
- A description of any restrictions on data availability
- For clinical datasets or third party data, please ensure that the statement adheres to our policy

he mass spectrometry proteomics data (raw files:.r aw, serach files: .msf, MGF and mzIdentML files exported from Scaffold, as well as Skyline files) have been deposited to the ProteomeXchange Consortium via the PRIDE partner repository with the project identifier PXD026486.

Human research participants

Policy information about studies involving human research participants and Sex and Gender in Research.

Reporting on sex and gender	n/a
Population characteristics	n/a
Recruitment	n/a
Ethics oversight	n/a

Note that full information on the approval of the study protocol must also be provided in the manuscript.

Field-specific reporting

Please select the one below that is the best fit for your research. If you are not sure, read the appropriate sections before making your selection.

- [x] Life sciences
- [ ] Behavioural & social sciences
- [ ] Ecological, evolutionary & environmental sciences

For a reference copy of the document with all sections, see nature.com/documents/nr-reporting-summary-flat.pdf

Life sciences study design

All studies must disclose on these points even when the disclosure is negative.

Sample size	Sample size was determined as a minimum of three replicates for in vitro experiments with recombinant protein. No power calculations were performed. We used n=3 technical replicates per sample as this is what was operationally feasible due to the simultaneous handling of multiple mutants. All experiments were repeated 2 additional times and pooled data shown.
Data exclusions	No data were excluded from the analysis.
Replication	All experiments were repeated a minimum of 3 times, with the exception of the mass spectrometry analysis which was replicated 2 times. Confirmation of consistency between replicates was ensured prior to pooling experiments.
Randomization	Randomization was not relevant to experiments conducted in this study, which consisted of mechanistic in vitro work.
Blinding	Blinding was not relevant to the experiments conducted in this study

Reporting for specific materials, systems and methods

We require information from authors about some types of materials, experimental systems and methods used in many studies. Here, indicate whether each material, system or method listed is relevant to your study. If you are not sure if a list item applies to your research, read the appropriate section before selecting a response.

Materials & experimental systems

n/a	Involved in the study	
[ ]	[x]	Antibodies
[x]	[ ]	Eukaryotic cell lines
[x]	[ ]	Palaeontology and archaeology
[x]	[ ]	Animals and other organisms
[x]	[ ]	Clinical data
[x]	[ ]	Dual use research of concern

Methods

n/a	Involved in the study	
[x]	[ ]	ChIP-seq
[x]	[ ]	Flow cytometry
[x]	[ ]	MRI-based neuroimaging

Antibodies

Antibodies used	Anti mouse glutaredoxin (GLRX) 1 antibody (Rockland)
Validation	This antibody was custom made for our laboratory by Rockland Immunochemicals (Limerick, PA, USA). Briefly, recombinant murine GLRX was used to generate a rabbit polyclonal antibody, which was affinity-purified and validated in Glrx knockout mouse lung tissue.

Validation of the antibody is provided in Supplementary Figure 11.
